# Affinity of Skp to OmpC revealed by single-molecule detection

**DOI:** 10.1038/s41598-020-71608-4

**Published:** 2020-09-10

**Authors:** Sichen Pan, Chen Yang, Xin Sheng Zhao

**Affiliations:** 1 Beijing National Laboratory for Molecular Sciences, State Key Laboratory for Structural Chemistry of Unstable and Stable Species, and Department of Chemical Biology, College of Chemistry and Molecular Engineering, Peking University, Beijing, 100871 China; 2 Biomedical Pioneering Innovation Center (BIOPIC), Peking University, Beijing, 100871 China

**Keywords:** Biophysics, Membrane biophysics, Single-molecule biophysics

## Abstract

Outer membrane proteins (OMPs) are essential to gram-negative bacteria, and molecular chaperones prevent the OMPs from aggregation in the periplasm during the OMPs biogenesis. Skp is one of the molecular chaperones for this purpose. Here, we combined single-molecule fluorescence resonance energy transfer and fluorescence correlation spectroscopy to study the affinity and stoichiometric ratio of Skp in its binding with OmpC at the single-molecule level*.* The half concentration of the Skp self-trimerization (*C*_1/2_) was measured to be (2.5 ± 0.7) × 10^2^ nM. Under an Skp concentration far below the *C*_1/2_, OmpC could recruit Skp monomers to form OmpC·Skp_3_. The affinity to form the OmpC·Skp_3_ complex was determined to be (5.5 ± 0.4) × 10^2^ pM with a Hill coefficient of 1.6 ± 0.2. Under the micromolar concentrations of Skp, the formation of OmpC·(Skp_3_)_2_ was confirmed, and the dissociation constant of OmpC·(Skp_3_)_2_ was determined to be 1.2 ± 0.4 μM. The precise information will help us to quantitatively depict the role of Skp in the biogenesis of OMPs.

## Introduction

The outer membrane (OM) in the gram-negative bacteria is crucial for bacterial survival by separating the periplasm and the external environment^1^. Most outer membrane proteins (OMPs) in the OM adopt a porin-like β-barrel conformation to take up nutrients and to excrete toxic waste products. Since a single OMP polypeptide is synthesized in the cytoplasm in an unfolded form, they have to be transported through the aqueous periplasm and to be folded in the OM, several periplasmic quality control factors, such as SurA, Skp and DegP, are involved in the protection of OMPs from mis-folding and aggregation^2–5^. Among those periplasmic quality control factors, Skp is shown to have the ability to break up the OMP aggregates^6^. It is shown that the accumulated OMP aggregates in the periplasm will stimulate the σ^E^ response^7^, which downregulates the OMPs expression^8^ and upregulates the Skp expression^9^.

The crystallographic analysis reveals that Skp has a “jellyfish”-like structure in the homo-trimer^10,11^, which makes people believe that Skp_3_ is the basic unit to interact with OMPs. Lyu et al. show that in the formation of the OMP-Skp complex the N-terminus of OMPs enters the cavity of Skp_3_ first and eventually the whole OMPs are engulfed^12^ via the electrostatic and hydrophobic interactions^13^. Additionally, the Skp_3_ tentacles are remarkably flexible to adjust the cavity size to accommodate OMPs of different sizes^14,15^.

Recently, the dynamic equilibrium between the Skp monomer and the Skp_3_ trimer under a physiological concentration was demonstrated^16^, while the affinity between Skp and OMPs is in the nanomolar range^13,17^. Therefore, it is speculated that the Skp monomer may be involved in the Skp chaperone activity^16^. Moreover, by using bulk kinetic fluorescence spectroscopy and mass spectrometry, Schiffrin et al*.* show that two Skp_3_ together can bind large OMPs^15^. Although much is learnt about the biophysical and biochemical properties of Skp, there are still certain theoretical and methodological issues that need to be addressed. On the one hand, OMPs tend to aggregate under the ensemble concentrations, which will complicate the interaction between OMPs and molecular chaperones. On the other hand, there are many subpopulations in the solution. For example, OMPs are in the equilibrium between the chaperone-unbound state and the chaperone-bound state^18^, and Skp is in the equilibrium between the Skp monomer and the Skp_3_ trimer^16^. These equilibria will blur the information at the ensemble level. For instance, it is unclear what is exactly the stoichiometric ratio in the OMP-Skp complex when the Skp and OMP monomers are predominant in the solution. Meanwhile, some affinity constants of the OMP-Skp complexes are still unknown.

Here, we implemented a few tactics that combine single-molecule fluorescence resonance energy transfer (smFRET) and fluorescence correlation spectroscopy (FCS) to overcome the aforementioned obstacles^19^. We picked out a portion of the smFRET components and carried out an FCS study on the molecular weight of the selectively chosen species. To call our treatment conveniently, we named it the portion-selectively-chosen fluorescence correlation spectroscopy (pscFCS). Unlike some other separation and identification methods, smFRET and FCS does not perturb the molecular interactions and equilibria. Furthermore, smFRET may prepare and investigate OMP samples in the monomeric form, so that the interference of OMP aggregation can be completely removed^6^. Then, FCS allows us to determine the molecular weight of very dilute samples so as to derive the stoichiometric ratio under very low concentrations. We used smFRET, pscFCS and amine-crosslinked sodium dodecyl-sulfate polyacrylamide gel electrophoresis (SDS-PAGE) to study the affinity and the stoichiometric ratio in the OmpC-Skp complexes. We found that OmpC induced the trimerization of Skp to form OmpC·Skp_3_ when the Skp concentration was far below its self-trimerized concentration. When the Skp concentration reached the micromolar range, another Skp_3_ can bind with OmpC·Skp_3_ to form OmpC·(Skp_3_)_2_. The affinity constants of relevant processes were quantitatively determined. Our results provided new information to help better understanding the role of Skp in the safe-guarding and quality control of OMPs in the periplasm.

## Results

### Development of pscFCS for specific subpopulations

The protection of OMPs by Skp involves many different states of OMPs and Skp. To study a specific subpopulation, we developed pscFCS which used both smFRET and FCS. Namely, we measured the smFRET histogram, from which the fluorescence traces of a specific subpopulation were selected and the FCS curves were calculated (SI methods).

**Figure 1:**
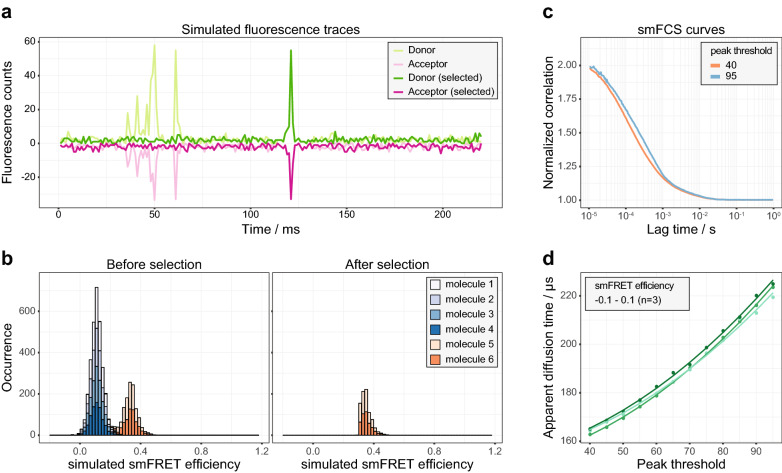
The procedure to yield the unbiased diffusion time for a specific subpopulation from pscFCS. (**a**) The fluorescence bursts of interest were selected and the rest part was replaced by the generated Poisson noises. The donor fluorescence bursts before (light green) and after (green) the selection were plotted. The acceptor fluorescence bursts multiplied by -1 before (light magenta) and after (magenta) the selection were plotted. (**b**) the smFRET histogram of the originally simulated fluorescence traces showed two smFRET efficiency peaks at the position of 0.11 and 0.33, respectively. After the selection of the fluorescence traces in the smFRET efficiency range of 0.3–0.5, the recalculated smFRET histogram only showed the smFRET efficiency peak within 0.3–0.5, which was mainly contributed by the molecules 5 and 6. (**c**) The peak threshold affected the apparent diffusion time of the pscFCS curves for Cy3B. The larger the peak threshold was, the larger the apparent diffusion time out of the pscFCS curve. (**d**) The apparent diffusion time showed a non-linear relationship to the peak threshold. The dots and the lines represent the experimental data and the fitted curves, respectively. Data are shown of three independent experiments.

Figure 1a shows typical simulated fluorescence traces under a condition of single-molecule detection (SMD). The fluorescence traces within a certain range of smFRET efficiencies were retained and the rest were replaced by the generated Poisson noises. Figure 1b shows the smFRET histograms before and after the selection of the simulated fluorescence traces. In the simulation, 6 molecules diffused freely through the laser focus, with four of them (labelled as molecule 1–4) having a theoretical smFRET efficiency of 0.11 and two of them (labelled as molecule 5 and 6) having a theoretical smFRET efficiency of 0.33. Therefore, the smFRET efficiency between 0.3 and 0.5 was mainly contributed by the molecules 5 and 6. The fluorescence traces in that smFRET efficiency range were selected to recalculate the smFRET histogram. Consequently, the recalculated smFRET histogram contained only the smFRET signals from the molecules 5 and 6, while the contribution of other molecules was removed.

The dynamic process between the donor and acceptor may alter the FCS curve. In order to get rid of this effect, the subpopulation-specific traces of the donor and acceptor channels were added together to cancel the anti-correlation caused by the dynamic process between the donor and acceptor (Supplementary Figs. S1 and S2). Then, the synthesized traces were used to calculate the FCS curves. A 2-dimensional diffusion (2D) model was used to fit the FCS curve within the time range of 10 μs to 1 s to obtain the apparent diffusion time of a specific subpopulation (SI methods),

1 $$G\left( t \right) = \frac{{\left\langle {I\left( 0 \right)I\left( {\text{t}} \right)} \right\rangle }}{{\left\langle {I\left( {\text{t}} \right)} \right\rangle^{2} }} = 1 + \frac{{G_{0} }}{{\left( {1 + t/\tau_{{{\text{app}}}} } \right)}}$$

where *I* is the fluorescence intensity, $\tau_{{{\text{app}}}}$ is the apparent diffusion time and $G_{0}$ is the inverse of the number of fluorescent molecules in the laser focus.

In the pscFCS treatment, a peak threshold was set to pick out fluorescence signals from the background noises. The larger the peak threshold was, the more the bursts of low photon counts were disregarded. Obviously, the peak threshold would generate a bias on the apparent diffusion time of the pscFCS curve. To find a way to acquire the unbiased diffusion time, we chose Cy3B as a standard sample to calculate the pscFCS curves at different peak thresholds and to investigate the relation between the apparent diffusion time and the peak threshold. Cy3B has only the zero-efficiency peak in the smFRET histogram (Supplementary Fig. S3). Thus the fluorescence traces with the smFRET efficiencies of − 0.1 to 0.1 were selected to calculate the pscFCS curves and to extract the apparent diffusion time (Supplementary Figs. S4–S8). The pscFCS curves at two different peak thresholds are shown in Fig. 1c, and the relation between the apparent diffusion time and the peak threshold of three parallel experiments is shown in Fig. 1d. We empirically found that the relation between the apparent diffusion time and the peak threshold can be fitted by

2 $$\tau_{{{\text{app}}}} = \tau+ ax^{2}$$

where $x$ is the peak threshold, *τ* and *a* are fitting parameters. Equation (2) was used to extrapolate the curve to the y-axis to get the unbiased diffusion time *τ*. As a verification, the* τ* of Cy3B measured by the pscFCS was 151 ± 2 μs (n = 3, Fig. 1d), and the standard diffusion time of Cy3B measured by the conventional FCS was 162 ± 8 μs (n = 5, Supplementary Fig. S9). The relative error was 6.8%, which was sufficient for us to identify the stoichiometric ratio in the OmpC-Skp complex.

The molecular weight was derived from the diffusion time. According to the Stokes–Einstein equation, the diffusion coefficient $D$ of a spherical particle in a liquid with a low Reynolds number is

3 $$D = \frac{{k_{{\text{B}}} T}}{6\pi \eta R}$$

where *k*_B_ is the Boltzmann’s constant, *T* is the temperature, $\eta$ is the dynamic viscosity, and $R$ is the hydrodynamic radius. $D$ is related to the diffusion time $\tau$ by

4 $$\tau = \frac{{\omega_{{{\text{xy}}}}^{2} }}{4D}$$

where $\omega_{{{\text{xy}}}}$ is the waist of the laser beam in the $xy$ plane. To get the molecular weight from *D* by the Stokes–Einstein equation, we made three reasonable assumptions: (1) Proteins are in spherical shape, (2) proteins are in a simple solution so that the Stokes–Einstein equation holds, and (3) all protein species have the same molecular density. Under above assumptions, the following relation holds between two protein species with one of them taken as a reference,

5 $$\frac{{M_{1} }}{{M_{2} }} = \left( {\frac{{\tau_{1} }}{{\tau_{2} }}} \right)^{3}$$

where $M_{1}$, $M_{2}$, $\tau_{1}$ and $\tau_{2}$ are the respective molecular weights and diffusion times. The error propagation from the diffusion time to the molecular weight was derived to be

6 $$\frac{{{\upsigma }^{2} \left( {M_{1} } \right)}}{{\overline{{M_{1} }}^{2} }} = 9 \cdot \left( {\frac{{\sigma^{2} \left( {\tau_{1} } \right)}}{{\overline{{\tau_{1} }}^{2} }} + \frac{{\sigma^{2} \left( {\tau_{2} } \right)}}{{\overline{{\tau_{2} }}^{2} }}} \right)$$

### Formation of OmpC·Skp_3_ complexes under subnanomolar Skp concentrations

**Figure 2:**
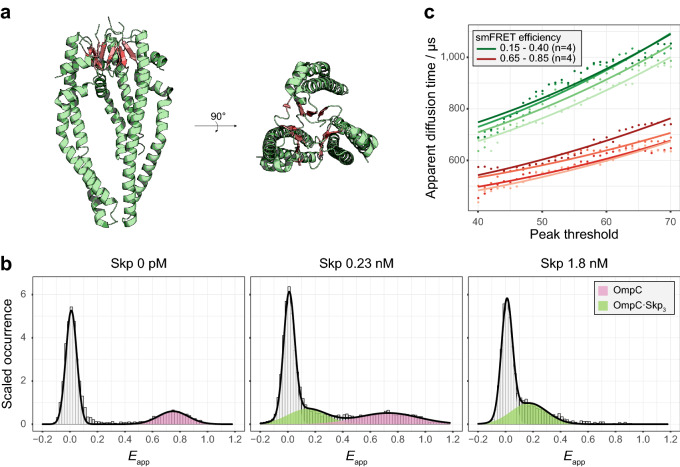
The observation of OmpC·Skp_3_ complexes under subnanomolar Skp concentrations. (**a**) the crystal structure of Skp_3_ (PDB ID: 1SG2^10^) was shown in the side-view and top-view. The β-sheets of each monomer are coloured in salmon, which form the trimeric core domain. (**b**) The smFRET histograms showed the conformational changes of OmpC G8C-D335C when it was incubated with Skp. These smFRET histograms exhibited two peaks of smFRET efficiency at 0.13 (green) and 0.73 (pink), which represented the bound- and unbound-OmpC, respectively. All histograms were normalized and fitted by the Gaussian distributions. (**c**) The apparent diffusion times of the bound- (green, smFRET efficiency ranges from 0.15 to 0.40) and unbound- (red, smFRET efficiency ranges from 0.65 to 0.85) OmpC at different peak thresholds were shown. The unbiased diffusion time of the bound-OmpC was 549 ± 27 μs and that of the unbound-OmpC was 422 ± 26 μs. The dots and the lines represent the experimental data and the fitted curves, respectively. Data are shown of four independent experiments.

The “jellyfish”-like structure of Skp_3_ consists of three subunits (Fig. 2a). We first studied the self-trimerization of Skp by the conventional FCS (SI methods and Supplementary Figs. S10−S12). The dissociation constant $K$ of Skp_3_ was measured to be (4.6 ± 2.7) × 10^4^ nM^2^, corresponding to the half concentration of the Skp self-trimerization ($C_{1/2}$) being (2.5 ± 0.7) × 10^2^ nM, which was read out from Supplementary Fig. S12. Our measured $C_{1/2}$ is smaller than but on the same order of magnitude with the reported $C_{1/2}$ of (4.4 ± 1.3) × 10^2^ nM under the most similar temperature and salt concentration^16^.

To investigate the stoichiometric ratio in the OmpC-Skp complex under very low Skp and OmpC concentrations, double mutant OmpC G8C-D335C was labelled with fluorescent dyes AF555 and AF647. We incubated the dual-labelled OmpC with wild-type Skp of various concentrations and carried out the pscFCS experiments. Because the smFRET efficiencies reflect the conformational change of OmpC between the unbound-state and the bound-state^6,17^, different apparent smFRET efficiencies (*E*_app_) correspond to different subpopulations. Figure 2b shows the smFRET histograms of OmpC in the presence of Skp. The peak at *E*_app_ = 0 (the zero-efficiency peak) was due to the species which did not have an acceptor or had an inactivated acceptor, and thus was disregarded. When Skp was absent, only the *E*_app_ = 0.78 peak was observed. At Skp concentration of 0.23 nM, an smFRET efficiency peak at *E*_app_ = 0.13 was observed besides the 0.78 peak. When Skp was 1.8 nM, only the 0.13 peak remained (more results in Supplementary Fig. S13). The 0.78 peak was assigned to unbound-OmpC and the 0.13 peak to bound-OmpC as previously observed^6^. Then, we used pscFCS to compare the diffusion time of the unbound- and bound-OmpC in the same smFRET histogram (Fig. 2c). The stoichiometric ratio $r = {\text{Skp}}:{\text{OmpC}}$ was derived to be

7 $$r = \left( {\left( {\frac{{\tau_{{{\text{bound}}}} }}{{\tau_{{{\text{apo}}}} }}} \right)^{3} - 1} \right) \times \frac{{M_{{{\text{OmpC}}}} }}{{M_{{{\text{Skp}}}} }}$$

and was determined to be 2.8 ± 0.4 by using the unbound OmpC as the inner reference. The result showed that OmpC was already bound by Skp_3_ even though the concentration of Skp in the solution was much lower than the $C_{1/2}$ of the Skp self-trimerization.

**Figure 3:**
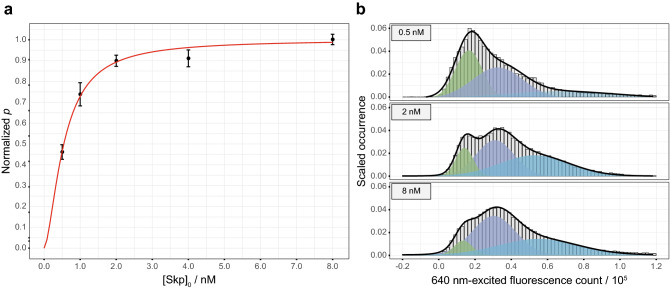
The quantification of affinity for the formation of OmpC⋅Skp_3_. (**a**) The apparent dissociation constant was obtained by fitting Supplementary Eq. (19) to the normalized event counts of colocalized donor–acceptor pairs in the TIRF experiments (normalized *p*) as a function of the total Skp concentration ([Skp]_0_). The black dots and the red line represent the experimental data and the fitted curve, respectively. Data are shown as mean ± s.d. of six imaging regions. (**b**) The histograms of the direct-excited acceptor fluorescence counts exhibited three peaks at 0.15 × 10^5^ (green), 0.31 × 10^5^ (indigo) and 0.56 × 10^5^ (azure) respectively. All histograms were normalized and fitted by the Gaussian distributions.

To accurately quantify the affinity between Skp and OmpC, we used total internal reflection fluorescence (TIRF) microscopy for a colocalization measurement, in which OmpC G8C-AF555 was immobilized on a glass surface and was incubated with freely diffusing Skp D128C-AF647. The apparent dissociation constant ($K_{{\text{D}}}$) for the reaction

8 $${\text{OmpC}} \cdot {\text{Skp}}_{3} \mathop \leftrightarrow \limits^{{K_{{\text{D}}} }} {\text{OmpC}} + 3{\text{Skp}}$$

was equal to the Skp concentration when half of the dye-labelled OmpC was in the OmpC-Skp complex, and it was determined to be (5.5 ± 0.4) × 10^2^ pM with a Hill coefficient $n_{{{\text{Hill}}}} = 1.6 \pm 0.2$ (Fig. 3a and SI method). Our measured $K_{{\text{D}}}$ is smaller than the reported apparent dissociation constant of Skp to OmpC^17^, probably due to the high sensitivity of the SMD method. The histograms of the acceptor fluorescence counts directly excited by the 640 nm laser showed three Gaussian peaks (Fig. 3b and Supplementary Fig. S14), which was consistent with the result of the pscFCS study, i.e., there were 3 Skp monomers in the OmpC-Skp complex.

### Formation of OmpC·(Skp_3_)_2_ complexes under micromolar Skp concentrations

**Figure 4:**
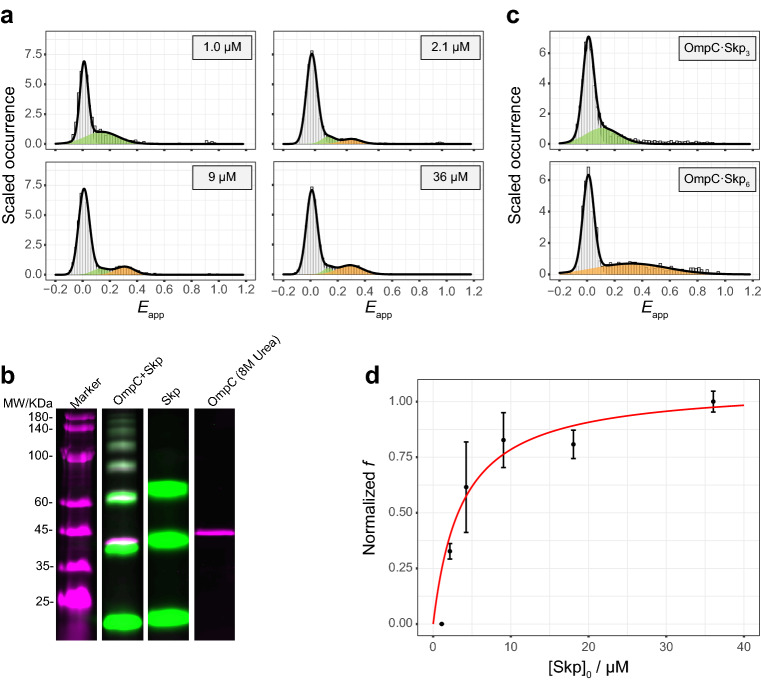
The observation of OmpC·(Skp_3_)_2_ complex under micromolar Skp concentrations. (**a**) As Skp concentration increased, the smFRET histogram of OmpC G8C-D335C showed that the peak of *E*_app_ = 0.1 (green) decreased and the peak of *E*_app_ = 0.3 (amber) increased. All the histograms were normalized and fitted by the Gaussian distributions. (**b**) OmpC-AF647 (magenta) and Skp-Cy3B (green) were incubated, crosslinked, and separated by the fluorescent SDS-PAGE, which showed six colocalized gel bands of OmpC·Skp_n_ complexes (n = 1–6). In the negative control of Skp or OmpC alone, no intermolecularly crosslinked product was observed. In Supplementary Fig. S18 the full image is presented. (**c**) The intramolecularly labelled OmpC G8C-D335C was incubated with the wild-type Skp and the gel bands of OmpC·Skp_3_ and OmpC·Skp_6_ were excised and retrieved back into the buffer to measure their smFRET efficiencies. The smFRET efficiency peaks of OmpC·Skp_3_ (green) and OmpC·Skp_6_ (amber) were identical to that under the in situ condition, respectively. All the histograms were normalized and fitted by the Gaussian distributions. (**d**) The normalized fraction of [OmpC·(Skp_3_)_2_] over [OmpC·Skp_3_] + [OmpC·(Skp_3_)_2_] (normalized *f*) as a function of [Skp]_0_ was fitted by Supplementary Eq. (21). The black dots and the red line represent the experimental data and the fitted curve, respectively. Data are shown as mean ± s.d. of three experiments.

As is shown in Fig. 4a and Supplementary Fig. S13, when the intramolecular labelled OmpC G8C-D335C was incubated with micromolar concentrations of Skp, the *E*_app_ = 0.3 peak appeared next to the previous peak of *E*_app_ = 0.1 in the smFRET histogram. The phenomenon was checked among different OmpC double mutants (Supplementary Figs. S15–S17). Previous study pointed out that Skp_3_ was multivalent^15^, so an intuitive hypothesis was that the conformational change of OmpC under the micromolar concentrations of Skp was due to the change in the stoichiometric ratio in the OmpC-Skp complexes. To verify the hypothesis, OmpC was incubated with Skp and separated in SDS-PAGE after an amine-crosslinking (Fig. 4b and Supplementary Fig. S18). So the molecular weights of the crosslinked products could be estimated from the SDS-PAGE bands. As shown in Fig. 4b, when OmpC G8C and Skp D128C were labelled by fluorescent dyes, six colocalized gel bands of Cy3B and AF647 from 60 to 180 kD were observed. The control experiments showed that Skp_3_ could be crosslinked intramolecularly but not intermolecularly when only Skp was present, and when only OmpC was present in an 8 M urea buffer, no intermolecularly crosslinked product was observed. The six colocalized gel bands were assigned to from OmpC·Skp to OmpC·Skp_6_ respectively according to their molecular weights (40.2 kD for His-tagged OmpC G8C-AF647 and 18.8 kD for His-tagged Skp D128C-Cy3B). The molecular weight determined by each identified gel band was slightly greater than the theoretical molecular weight due to the association of the DSS molecules (Supplementary Table S1). Because of the false negative and false positive possibilities, the crosslinked products did not necessarily represent the real subpopulations in the solution, but it suggested the possible existence of OmpC·Skp_6_ in the solution. To identify the origin of the *E*_app_ = 0.3 peak in the smFRET histograms, intramolecular labelled OmpC G8C-D335C was crosslinked with the wild-type Skp (Supplementary Fig. S19) and the gel bands of OmpC·Skp_3_ and OmpC·Skp_6_ were excised and retrieved back into the buffer to measure their smFRET efficiencies (Fig. 4c). The smFRET histograms showed that the retrieved OmpC·Skp_3_ had an *E*_app_ peak at 0.1 and the retrieved OmpC·Skp_6_ had an *E*_app_ peak at 0.3, consistent with the observed peaks under the in situ condition (Fig. 4a). The result confirmed the hypothesis that the OmpC conformational change under the micromolar concentrations of Skp was due to the change in the stoichiometric ratio of the OmpC-Skp complexes. By fitting the peak area in the smFRET histograms under the in situ condition against the [Skp]_0_, the dissociation constant $K_{{\text{D}}}^{^{\prime}}$ for the reaction

9 $${\text{OmpC}} \cdot ({\text{Skp}}_{3} )_{2} \mathop \leftrightarrow \limits^{{K_{{\text{D}}}^{^{\prime}} }} {\text{OmpC}} \cdot {\text{Skp}}_{3} + {\text{Skp}}_{3}$$

was determined to be $K_{{\text{D}}}^{^{\prime}}$ = 1.2 ± 0.4 μM (Fig. 4d and SI method).

Interestingly, the pscFCS study determined that the hydrodynamic radius of OmpC·(Skp_3_)_2_ was comparable to that of OmpC·Skp_3_ (Supplementary Fig. S20 and Supplementary Table S2), indicating that OmpC·(Skp_3_)_2_ adopts an “inter-locked” configuration (see discussion section for the detail).

## Discussion

We made a thorough study on the equilibrium and the stoichiometric ratio of the Skp self-trimerization and the Skp-OmpC binding reactions. In our experiments, the fluorescent dye was labelled on Skp D128C. Since both the dye and His-tag are much smaller than the protein and are distant from the key functional sites, a manageable effect is expected. Our previous study confirms that the dye labelling and His-tag attachment do not perturb the structure and chaperone activity^12,17^. The *C*_1/2_ of Skp self-trimerization was 250 nM, which is comparable to previous measurement^16^. So, the agreement on the equilibrium constant of the Skp self-trimerization also indicates that the attached dye had a negligible effect on the Skp trimerization.

In the OMPs biogenesis, OMP polypeptides are synthesized by ribosomes in the cytoplasm and secreted in an unfolded state into the periplasm through the SecYEG/SecA translocon. In the periplasm the OMP polypeptides are safeguarded by chaperones including Skp^1,20–23^. We were able to resolve individual subpopulations, and we experimentally proved that the OmpC⋅Skp_3_ complex was formed under the Skp concentrations far below the *C*_1/2_ of the self-trimerization. The positive cooperativity (*n*_Hill_ = 1.6 ± 0.2) for the OmpC⋅Skp_3_ formation suggests that the Skp monomer plays an important role in the OmpC·Skp_3_ formation when Skp monomer is the predominant form in the solution. The binding of Skp to OmpC is almost a diffusion-controlled reaction^24^, so the association rate constant between molecules A and B could be estimated by

10 $$k^{ + } = 4{\uppi }\left( {D_{{\text{A}}} + D_{{\text{B}}} } \right)\left( {R_{{\text{A}}} + R_{{\text{B}}} } \right)$$

where $D$ is the diffusion coefficient, and $R$ is the radius of molecules. The radius of Skp_3_ in a solution is 3.3 nm^25^, and based on our FCS data, the radius of Skp is 2.3 nm. According to Eqs. (3) and (10), the association rate constant of Skp to OmpC is $k_{{{\text{Skp}}}}^{ + }$ = 6.7 × 10^9^ M^−1^ s^−1^, and Skp_3_ to OmpC is $k_{{{\text{Skp}}_{3} }}^{ + }$ = 6.6 × 10^9^ M^−1^ s^−1^. When the equilibrium between Skp and Skp_3_ is considered, the apparent reaction rate constants are defined as $k_{{{\text{Skp}}}}^{ + } \left[ {{\text{Skp}}} \right]$ and $k_{{{\text{Skp}}_{3} }}^{ + } \left[ {{\text{Skp}}_{3} } \right]$. Consequently, $k_{{{\text{Skp}}}}^{ + } \left[ {{\text{Skp}}} \right]$ is several orders of magnitude larger than $k_{{{\text{Skp}}_{3} }}^{ + } \left[ {{\text{Skp}}_{3} } \right]$ when Skp concentration is in the submicromolar range. With the increase of the Skp concentration, $k_{{{\text{Skp}}_{3} }}^{ + } \left[ {{\text{Skp}}_{3} } \right]$ becomes progressively predominant (Supplementary Fig. S21). Therefore, the induced Skp trimerization by OmpC should be predominant under low Skp concentrations, while the direct reaction between Skp_3_ and OmpC becomes the major route when the concentration of Skp_3_ exceeds a certain level. Under even higher Skp concentrations, OmpC·(Skp_3_)_2_ can form.

The periplasm is lack of ATP as the energy source. The free energy of the OMP folding is from − 18 to − 32 kcal mol^−1^, which acts as an energy sink in the OMPs biogenesis^26^. Skp and SurA are two major OMPs’ chaperones in the periplasm. It is interesting to compare their binding property to OMPs. OMPs bind SurA by attaching to the SurA surface like a random coil and the binding affinity is mild^27^. These characters are associated with the role of SurA as the primary cargo in the OMPs’ delivery to the BAM complex^27^. Skp, on the other hand, holds OMPs in its pocket with a very strong affinity. Our results reveals that the free energy of Skp_3_ binding to OmpC is − 20 kcal mol^−1^, which supports the idea that Skp_3_ is a superior holdase^28^ and de-aggregation agency^6^ of OMPs. With such a large binding energy, the release of substrates from Skp may require the assistance of LPS^29,30^, DegP^2,31^ or the BAM complex^32,33^ to ensure the direction of the journey of OMPs towards OM. It is said that besides SurA, Skp and DegP together constitute another route to deliver OMPs to OM^2,31^. To figure out the molecular mechanism on the collaboration between Skp and DegP is deemed to be a very interesting topic for a future study.

**Figure 5:**
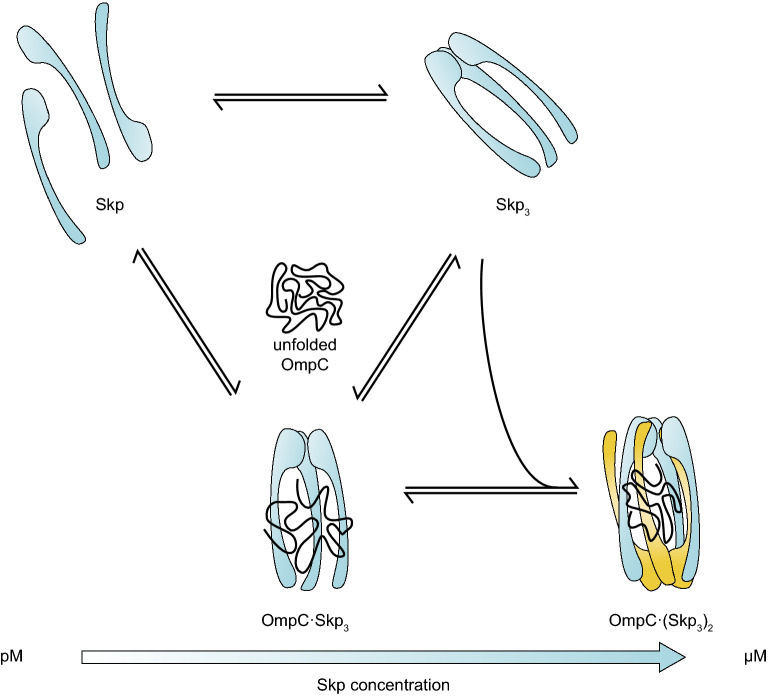
The schematic view of Skp binding OmpC. OmpC collapses in aqueous solution without chaperones. Skp binds OmpC to form OmpC·Skp_3_ complex under subnanomolar concentrations. Under submicromolar concentrations, Skp_3_ directly reacts with OmpC to form OmpC·Skp_3_. When Skp concentration is in the micromolar range, OmpC·(Skp_3_)_2_ will appear with an “inter-locked” binding pattern.

The formation of OmpC·(Skp_3_)_2_ was observed. When unbound-state Skp_3_ in the solution was used as a reference, we found that the hydrodynamic radius of OmpC·Skp_3_ is larger than that of Skp_3_ (Supplementary Fig. S20 and Supplementary Table S2). According to our pscFCS results, the hydrodynamic radius of OmpC·(Skp_3_)_2_ was similar to that of OmpC·Skp_3_ (Supplementary Table S2). This experimental result strongly suggested that the orientation of the two Skp_3_ in OmpC·(Skp_3_)_2_ is realized via the “inter-locked” pattern^15^. Our data showed that the intramolecular smFRET efficiency of the dual-labelled OmpC was higher in OmpC·(Skp_3_)_2_ than that in OmpC·Skp_3_ (Fig. 4, Supplementary Figs. S13 and S15–S17), indicating that OmpC was compressed tighter in OmpC·(Skp_3_)_2_ than in OmpC·Skp_3_, also consistent with the “inter-locked” model. The Skp physiological concentration of bacteria at stationary phase growth in LB is 2.1–3.9 μM^6,16^, which is close to the dissociation constant $K_{{\text{D}}}^{^{\prime}} = 1.2 \pm 0.4\,\upmu {\text{M}}$ for the second Skp_3_. The σ^E^ response will downregulate OMPs expression and upregulate expression of chaperones and proteases^7,9^. Although the whole OMPs biogenesis landscape will be much complicated in vivo, we speculate that the upregulated Skp will increase the population of OMP·(Skp_3_)_2_, which may enhance the protection of OMPs against aggregation, thus helping the cells to survive. Figure 5 presents a diagram how the stoichiometric ratio in the OmpC-Skp complex is proceeded as the Skp concentration varies. The stoichiometric modulation of different complex forms may play a distinct role in the OMPs biogenesis under different situations.

## Methods

### Protein expression, purification and mutagenesis

The pET28a vectors carrying relevant Skp, including an N-terminal His-tag, were transformed into *E. coli.* BL21(DE3) pLysS cells (TransGen Biotech). The cells were grown in LB medium containing 50 μg/mL kanamycin at 37 °C with shaking 220 rpm until the culture reached an OD_600_ of ~ 0.6 (after ~ 3 h). The culture was induced with 0.5 mM IPTG, expressed for 4 h and harvested by centrifugation. The cell pellet was resuspended in 80 mL buffer A (50 mM PB, 500 mM NaCl and 10 mM imidazole, pH = 8.0) and lysed by ultrasonication. The supernatant was collected by centrifugation at 20,000 g for 30 min, filtered by 0.22 μm syringe filters and loaded on the Ni–NTA (5 mL) column (GE Healthcare) equilibrated with buffer A. The column was washed extensively by buffer A and eluted with a gradient (25%, 100%) of buffer B (50 mM PB, 500 mM NaCl and 250 mM imidazole, pH = 8.0). The protein fractions were dialyzed against buffer C (50 mM PB and 100 mM NaCl, pH = 7.0) by the HiTrap desalting columns (GE Healthcare), and concentrated by the centrifugal filter units (Amicon). The aliquots were snap-frozen in the liquid nitrogen and stored in − 80 °C. For the purification of OmpC, the plasmids including an N-terminal His-tag were transformed into the *E. coli.* BL21(DE3) cells (TransGen Biotech) and 8 M urea was included in buffers A–C to prevent aggregation. For the immobilization, an AviTag (GLNDIFEAQKIEWHE) was introduced to the C-terminus of OmpC G8C. The Skp concentration was determined by the BCA protein assay kit (Pierce), according to the manufacturer’s instruction, and was recorded using Skp monomer as the unit. The OmpC concentration was determined by using the molar extinction coefficient of 65,210 M^−1^ cm^−1^ at 280 nm.

The Fast Mutagenesis System (TransGen Biotech) with the plasmids pET28a-*skp* and pET28a-*ompC* as templates was used to construct the site-directed cysteine mutants of Skp and OmpC according to the manufacturer’s instruction. For the Skp self-trimerization, Skp D128C was generated. For the intramolecular smFRET assays, OmpC G8C-L139C, OmpC G8C-I232C and OmpC G8C-D335C were generated. For the TIRF colocalization assays, OmpC G8C with an AviTag on the C-terminus was generated.

### Fluorescent dye labelling

The labelling of site-directed cysteine mutants of Skp and OmpC was performed as follows. tenfold molar excess of tris-2-carboxyethyl-phosphinie (TCEP) was used in proteins of 100 μM at 27 °C for 30 min to reduce disulfide bonds. fivefold molar excess of maleimide fluorescent dyes Cy3B (GE Healthcare), AF555 (Life Technologies) or AF647 (Life Technologies) was added and the solution was kept in the dark at 27 °C for 3 h. The excess dyes were removed by the HiTrap desalting columns (GE Healthcare) in buffer C. 4 M urea was included in the labelling and eluting buffer for Skp to disassemble the trimers and to expose the cysteine residues. 8 M urea was included in the labelling and eluting buffer for OmpC to prevent the aggregation. The absorbance of Cy3B (130,000 M^−1^ cm^−1^), AF555 (158,000 M^−1^ cm^−1^) and AF647 (239,000 M^−1^ cm^−1^) was used to determine the fluorescent dye concentration. For all samples, the extent of the labelling was near 90%.

### Biotinylation of OmpC

The bioitinylation of OmpC was performed by enzyme BirA, which can specifically recognize the AviTag’s lysine side chain and attach a biotin. Biotinylation reaction was performed in accordance with the instruction provided by the BirA enzyme kit (GeneCopoeia). The reaction was conducted by mixing 40 μM dye-labelled OmpC with an AviTag, 50 μM D-Biotin, 10 mM ATP, 10 mM MgOAc and 45 U/μL BirA in buffer C containing 4 M urea at 30 °C for 1 h. After the reaction the aliquots were snap-frozen in liquid nitrogen and stored in − 80 °C.

### Monte-Carlo simulation

The Monte-Carlo simulation was performed as described previously^34,35^ and simplified by putting 6 diffusing molecules into the box. Each fluorescence trace was tracked and calculated to obtain the smFRET histograms in which the contribution from each molecule was known. Then, all the fluorescence traces were connected together to select the fluorescence bursts of certain subpopulations according to the smFRET efficiency. The synthesized fluorescence traces were used to recalculate the smFRET histograms to test whether the contribution of other subpopulations was removed.

### FCS measurement

The titration on the self-trimerization of Skp was carried out with a home-built inverted fluorescence confocal microscope based on a TE2000-U microscope (Nikon) equipped with a 532 nm solid-state laser (MLL-III-532-20mW, LD&TEC) as previously described^17,36^. The laser was adjusted to 100 μW and focused inside the sample solution through an oil immersion objective (NA 1.45, 60×, Nikon). The fluorescence was separated from the excitation light by a dichroic mirror (zt532 rdc, Chroma). After being focused through a 50 μm pinhole, the fluorescence was separated by a polarizing beam splitter (PBS) (China Daheng) into a reflected s-polarized beam and a transmitted p-polarized beam. The beams were focused on two avalanche photon diodes (APD) (SPCM-AQRH-14, PerkinElmer Optoelectronics) respectively for the FCS measurement. For each sample, Skp D128C-Cy3B of 40 nM was added in the wild-type Skp of various concentrations and the sample was denatured by 4 M urea. The solution was diluted 100-fold in buffer C and incubated in the dark at 24 °C for 30 min. The final concentration of Skp D128C-Cy3B was 0.4 nM. The 40 μL solution was sealed between a Secure-Seal hybridization chamber gasket (Life Technologies) and a cover glass. Experiments were conducted at 23 °C. The surface adsorption of proteins was prevented by 0.02% (v/v) tween 20 (Surfact-Amps 20, Life Technologies) in the buffer. The diffusion time $\tau$ of the sample was obtained by fitting the measured FCS curve using a formula considering 2-dimentional diffusion plus one exponential relaxation (2D1R):

11 $$G\left( t \right) = 1 + \frac{{G_{0} }}{{\left( {1 + t/\tau } \right)}} \times \left( {1 + A \cdot \exp \left( { - \frac{t}{{t_{{\text{R}}} }}} \right)} \right)$$

where $\tau$ is the diffusion time, $t_{{\text{R}}}$ is the relaxation time, $G_{0}$ is the inverse of the number of the fluorescent molecules in the laser focus and $A$ is the amplitude of the relaxation. The $\tau$ was used to calculate the effective Skp stoichiometric number $n$ according to the Stokes–Einstein equation, where the diffusion time and molecular weight of Cy3B were used as a reference:

12 $$n = \left( {\frac{{\tau_{{\text{D}}} }}{{\tau_{{{\text{Cy}}3{\text{B}}}} }}} \right)^{3} \cdot \frac{{M_{{{\text{Cy}}3{\text{B}}}} }}{{M_{{{\text{Skp}}}} }}$$

where $M_{{{\text{Cy}}3{\text{B}}}}$ and $M_{{{\text{Skp}}}}$ are the molecular weight of Cy3B and Skp, and $\tau_{{{\text{Cy}}3{\text{B}}}}$ is the diffusion time of Cy3B. The $n$ as a function of [Skp]_0_ was fitted by Supplementary Eqs. (11)–(15) to obtain the dissociation constant for the Skp self-trimerization.

### smFRET measurement

The smFRET experiments were carried out by using the same confocal microscope as that for the Skp self-trimerization study. After the pinhole, the fluorescence was divided by a dichroic mirror T635 lpxr (Chroma) into the donor and acceptor channels. BrightLine 593/40 nm (Semrock) was put before the APD in the donor channel and HQ 685/40 nm (Chroma) was put before the APD in the acceptor channel. The 40 μL solution was sealed between a Secure-Seal hybridization chamber gasket (Life Technologies) and a cover glass. For the intramolecular smFRET study, 8 M urea denatured dual-labelled OmpC was diluted into a buffer solution with the required Skp concentration, and the final concentration of OmpC was 50 pM. The samples were incubated in the dark for 15 min at the room temperature and the experiments were conducted at 23 °C. The surface adsorption of the proteins was prevented by 0.02% (v/v) tween 20 (Surfact-Amps 20, Life Technologies) in the buffer. The fluorescence of every sample was collected for 30 min in 1 ms bintime. The fluorescence data was processed by the Python scripts to yield the smFRET efficiency by

13 $$E_{{{\text{app}}}} = \frac{{I_{{{\text{Ac}}}} }}{{I_{{{\text{Dr}}}} + I_{{{\text{Ac}}}} }}$$

where $E_{{{\text{app}}}}$ is the apparent smFRET efficiency, $I_{{{\text{Dr}}}}$ and $I_{{{\text{Ac}}}}$ are the photon counts of the donor and acceptor of every identified fluorescence burst, respectively. The statistics of the smFRET efficiency yielded the smFRET histogram and the histogram was fitted by the Gaussian distributions.

### pscFCS measurement

The pscFCS operations were the same as that of the smFRET experiment except that the bintime was 0.96 μs. The data of every sample was processed by the Python scripts to obtain the unbiased diffusion time. More details about pscFCS is in the supplementary information.

### TIRF colocalization measurement

The PEG-passivated slides were prepared as previously described^37^. OmpC G8C-AF555 with an AviTag on the C-terminus was immobilized on a coverslip surface via the biotin-streptavidin interaction. A chamber anchored on the surface was incubated with 0.05 mg/mL streptavidin and then washed by buffer C. Afterwards, the 8 M urea denatured biotinylated OmpC was diluted 50-fold in buffer C to a final concentration of 50 pM and was added into the chamber. The chamber was washed again by buffer C. Finally, Skp D128C-AF647 with concentrations from 500 pM to 8 nM in buffer C was added into the chamber. The surface adsorption of the proteins was prevented by 0.02% (v/v) tween 20 (Surfact-Amps 20, Life Technologies) in the buffer and an oxygen scavenging system was included in the buffer during the detection^37^. The colocalization measurement was performed on a home-built TIRF microscope using alternating laser excitation (ALEX) between 532 and 640 nm at 23°C^37^. The emitted fluorescence from the molecules on the surface was separated by the filters and collected by a dual-view EMCCD with a frame frequency of 10 Hz. The colocalized fluorescence spots of the donor and acceptor were counted as described before^37^. The traces of the monomeric OmpC bound by Skp were selected to yield the histogram of the 640 nm-excited fluorescence counts and the apparent smFRET histogram. The apparent smFRET efficiency was calculated by Eq. (13).

### Amine-crosslinked fluorescent SDS-PAGE and retrieved smFRET measurement

For the amine-crosslinked fluorescent SDS-PAGE analysis, the samples were prepared by 50-fold dilution of OmpC G8C-AF647 into a mixture of wild-type Skp and Skp D128C-Cy3B of 0.9 μM in buffer C, and the final concentration of OmpC G8C-AF647 was 45 nM. Then the sample was incubated at room temperature in the dark for 15 min before adding the cross linker DSS (Thermo Fisher) with a final concentration of 20 μM. The sample was incubated for another 30 min, separated by SDS-PAGE and fluorescently imaged by Typhoon FLA 9,500 (GE Healthcare). For the retrieved smFRET experiments, the sample with dual-labelled OmpC and wild-type Skp was incubated and crosslinked with the same procedure as described above. Then, glycine was added to a final concentration of 10 mM to terminate the crosslinking reaction. The sample was concentrated by a centrifugal filter unit and separated by SDS-PAGE. The corresponding gel bands were excised and lysed in 200 μL buffer C and shaken at 4 °C, 220 rpm overnight. Then, the sample was centrifugated and the supernatant was purified by a centrifugal filter unit with tenfold volume of buffer C. Finally, the sample was diluted in buffer C to a single-molecule concentration to carry out the smFRET experiments.

## Supplementary information

Supplementary information.

## Data Availability

The datasets and code in the study are available in the github repository, https://github.com/psichen/affinity-of-Skp-to-OmpC.git.
