# Supplementary material for: Affinity of Skp to OmpC revealed by single-molecule detection
Source: Sci Rep. 2020 Sep 10;10:14871. doi: 10.1038/s41598-020-71608-4 (PMC7483523; doi:10.1038/s41598-020-71608-4)
Supplement: Supplementary file 1 — Supplementary information. [file 41598_2020_71608_MOESM1_ESM.pdf]

Supplementary information for:

Affinity of Skp to OmpC revealed by single-molecule detection

Sichen Pan, Chen Yang, Xin Sheng Zhao*

Beijing National Laboratory for Molecular Sciences, State Key laboratory for Structural Chemistry of Unstable and Stable Species, and Department of Chemical Biology, College of Chemistry and Molecular Engineering, Peking University, Beijing 100871, China.

Biomedical Pioneering Innovation Center (BIOPIC), Peking University, Beijing 100871, China.

* Email of correspondence: zhaoxs@pku.edu.cn

Supplementary Notes

Supplementary note 1: Data processing in pscFCS

First, the high-resolution (0.96 μs-bintime) fluorescence traces of the samples were recorded. The traces were binned to 1.008 ms bintime to generate the normal histogram of smFRET efficiency. Next, we added the original high-resolution fluorescence traces of the donor channel and the acceptor channel together, and selected the whole fluorescence bursts that belong to a specific subpopulation in the smFRET histogram. The rest in the original high-resolution fluorescence traces was replaced by the Poisson noises. Then the FCS curves were calculated by using the synthesized high-resolution traces and fitted to yield the apparent diffusion time of the specific subpopulation.

The fluorescence fluctuation due to the singlet-triplet transition or other relaxations may have an impact on extracting the diffusion time. To evaluate the effect due to the FRET process, we simulated 20 sets of two-component Brownian motion with an accompanying FRET process of different relaxation times. The cross correlation was not used since the cross correlation between the donor and acceptor has obvious anti-correlation components from the FRET process, which strongly interfere with the diffusion time. The autocorrelations of acceptor traces only, donor traces only, and donor + acceptor traces together were compared. The FCS curves were fitted by either a model of 2-dimentional diffusion with 1 relaxation term (2D1R),

$$G(t) = 1 + \frac{G_0}{(1+\frac{t}{\tau_{\mathrm{app}}})} \times (1 + A \cdot \exp\left(-\frac{t}{t_{\mathrm{R}}}\right)) \qquad (1)$$

or a model of 3-dimentional diffusion with 1 relaxation term (3D1R),

$$G(t) = 1 + \frac{G_0}{\left(1+\frac{t}{\tau_{\mathrm{app}}}\right)\left(1+\frac{t}{\tau_{\mathrm{app}}\cdot\omega^2}\right)^{\frac{1}{2}}} \times \left(1 + A \cdot \exp\left(-\frac{t}{t_{\mathrm{R}}}\right)\right) \quad (2)$$

where τ_{app} is the apparent diffusion time, t_{R} is the relaxation time, G_0 is the inverse of the number of fluorescent molecules in the laser focus, A is the amplitude for the relaxation and ω is the ratio of the beam waist in the z direction over the beam waist in the xy plane.

From the simulation we found that in the autocorrelation of acceptor traces or donor traces, the apparent diffusion time decreased when the relaxation exists, and the apparent diffusion time depended on specific relaxation dynamics of molecules. However, the autocorrelation of the donor + acceptor traces together was not influenced by the existence of the FRET relaxation (Supplementary Fig. S1). We also found that the 2D1R fitting yielded systematically smaller diffusion time than the theoretical values, whereas the diffusion time of the 3D1R fitting matched the theoretical values well. However, since we used the ratio on the diffusion time to derive the stoichiometric ratio of complexes, the difference in that ratio between the 2D1R and 3D1R models was not significant (Supplementary Fig. S2).

In the calculation of the smFRET histogram, a threshold on the fluorescence counts (named as the peak threshold) was taken to identify the qualified fluorescence bursts. Namely, the fluorescence bursts with a maximum photon counts below the peak threshold were disregarded. As the peak threshold increased, the proportion of fluorescence bursts with a high photon counts would increase in the smFRET histograms. The higher the photon counts

of fluorescence bursts was, the larger the apparent diffusion time of the FCS curve was, because the molecules were deeper in the Gaussian-shaped focus,

$$I = I_0 \mathrm{e}^{-2\left(\frac{\mathrm{x}^2}{\omega_{\mathrm{xy}}^2}+\frac{y^2}{\omega_{\mathrm{xy}}^2}+\frac{z^2}{\omega_{\mathrm{z}}^2}\right)} \tag{3}$$

where I_0 is the laser intensity at the center, ω_{xy} is the beam waist in the xy plane and ω_{z} is the beam waist in the z direction. Thus, an empirical polynomial equation was used to get the unbiased diffusion time,

$$\tau_{\mathrm{app}} = \tau + ax^2 \tag{4}$$

where x is the peak threshold, τ is the unbiased diffusion time and a is a fitting parameter. Our simulation demonstrated that the first-order derivative of τ_{app} at the origin was zero. Therefore, we did not include the linear term in the equation.

We exemplified Cy3B to examine which model is better to reduce the effect of the singlet-triplet transition (Supplementary Fig. S3). The transition usually occurs on the μs timescale, so we compared the results of the 10 μs-started FCS curves and 1 μs-started FCS curves by using the 2-dimensional diffusion with (2D1R model, equation (1)) or without (2D model, equation (5)) a relaxation term.

$$G(t) = 1 + \frac{G_0}{(1+t/\tau_{\mathrm{app}})} \tag{5}$$

For the 10 μs-started FCS curves, the singlet-triplet transition was mostly dropped out, so that the 2D model was suitable to fit (Supplementary Fig. S4) and yielded the apparent diffusion

time of 151±2 μs as shown in Fig. 1d in the main text. For the 1 μs-started FCS curves, the singlet-triplet transition was obvious, and the 2D model could not fit the curves well (Supplementary Fig. S5). The extrapolated unbiased diffusion time (Supplementary Fig. S6) was 143±2 μs. For the 2D1R model, although the 1 μs-started FCS curves could be well fitted (Supplementary Fig. S7), the apparent diffusion time was irregular due to large fitting errors (Supplementary Fig. S8). The standard diffusion time of Cy3B measured by the conventional FCS was 162±8 μs (Supplementary Fig. S9). Therefore, we chose to use the 2D model to fit the 10 μs-started pscFCS curves of the synthesized traces in our experiments.

Supplementary note 2: Derivation of dissociation constants of Skp self-trimerization

The Skp self-trimerization is described by

$$\mathrm{Skp}_3 \overset{K}{\leftrightarrow} 3\mathrm{Skp} \tag{6}$$

$$K = \frac{[\mathrm{Skp}]^3}{[\mathrm{Skp}_3]} \tag{7}$$

where K is the dissociation constant. When the dye-labelled Skp is mixed with far excessive wild-type Skp, the trimerization reaction with Skp^* as a component becomes

$$\mathrm{Skp}_3^* \overset{K^*}{\leftrightarrow} \mathrm{Skp}^* + 2\mathrm{Skp} \tag{8}$$

$$K^* = \frac{[\mathrm{Skp}^*][\mathrm{Skp}]^2}{[\mathrm{Skp}_3^*]} \tag{9}$$

where Skp^* is the dye-labelled Skp, Skp_3^* is the Skp trimer which contains one dye-labelled Skp monomer, and K^* is the respective dissociation constant. Because the wild-type Skp is

able to form trimer among themselves, reaction (8) and reaction (6) are coupled together.

The FCS experiments measured the effective diffusion time τ of the species containing dye-labelled Skp. Then, it can be converted to the molecular weight and then to the effective stoichiometric number of the detected Skp species (n), which is the average of the stoichiometric numbers of fluorescent Skp species,

$$n = n_{\mathrm{t}} \cdot \frac{[\mathrm{Skp}_3^*]}{[\mathrm{Skp}_3^*]+[\mathrm{Skp}^*]} + n_{\mathrm{m}} \cdot \frac{[\mathrm{Skp}^*]}{[\mathrm{Skp}_3^*]+[\mathrm{Skp}^*]} \tag{10}$$

where n_{t}=3 and n_{m}=1 by definition. Substituting equation (9) into equation (10) we have

$$n = n_{\mathrm{t}} \cdot \left(\frac{[\mathrm{Skp}]^2}{K^*+[\mathrm{Skp}]^2}\right) + n_{\mathrm{m}} \cdot (\frac{K^*}{K^*+[\mathrm{Skp}]^2}) \tag{11}$$

Since $[\mathrm{Skp}^*]<<[\mathrm{Skp}]$, the consumption of Skp by reaction (8) can be neglected and the mass balance can be written as

$$[\mathrm{Skp}]_0 = [\mathrm{Skp}] + 3[\mathrm{Skp}_3] \tag{12}$$

where $[\mathrm{Skp}]_0$ is the total concentration of the Skp. Combining equations (7) and (12), we derive that

$$[\mathrm{Skp}]^3 + \frac{K}{3} \cdot [\mathrm{Skp}] - \frac{K}{3} \cdot [\mathrm{Skp}]_0 = 0 \tag{13}$$

which has a single real root as

$$[\mathrm{Skp}] = \left(\frac{K}{6} \cdot [\mathrm{Skp}]_0 + (\Delta)^{\frac{1}{2}}\right)^{\frac{1}{3}} + \left(\frac{K}{6} \cdot [\mathrm{Skp}]_0 - (\Delta)^{\frac{1}{2}}\right)^{\frac{1}{3}} \tag{14}$$

where Δ is

$$\Delta = \left(\frac{\frac{K}{3}\cdot[\mathrm{Skp}]_0}{2}\right)^2 + \left(\frac{K}{9}\right)^3 \quad (15)$$

The n as a function of $[\mathrm{Skp}]_0$ can be fitted by substituting equation (14) into equation (11) to obtain both the dissociation constants K^* and K. The proportion of Skp in the trimer form to $[\mathrm{Skp}]_0$ (y) is

$$y = \frac{3[\mathrm{Skp}_3]}{[\mathrm{Skp}]_0} \quad (16)$$

Substituting equation (12) and equation (16) into equation (13), the relation between $[\mathrm{Skp}]_0$ and y is derived to be

$$[\mathrm{Skp}]_0 = \left(\frac{K}{3}\cdot\frac{y}{(1-y)^3}\right)^{\frac{1}{2}} \quad (17)$$

In the measurement of the equilibrium constant of the self-trimerization between Skp and Skp_3, we labelled fluorescent dye Cy3B to mutant Skp D128C. Skp D128C-Cy3B of 0.4 nM was mixed with the wild-type Skp ranging from 0 to 10^3 nM in buffer C (50 mM PB, 100 mM NaCl, pH 7.0). The FCS curve was recorded on a home-built confocal microscope and the data were fitted by the 2D1R model (Supplementary Fig. S10). The titration curve of n as a function of $[\mathrm{Skp}]_0$ was plotted and fitted by using above equations (Supplementary Fig. S11). This titration curve showed that the Skp concentration at which half Skp^* was monomer was 37 nM, and the derived dissociation constant for Reaction (8) was $K^*=(1.2\pm0.1)\times10^3$ nM^2. The dissociation constant for Reaction (6) was $K=(4.6\pm2.7)\times10^4$ nM^2, indicating that the

concentration at which half Skp molecules were in the trimer ($C_{1/2}$) was (2.5±0.7)×10^2 nM (Supplementary Fig. S12).

It is interesting to observe that different values of K and K^* were obtained. However, the deviation is merely superficial caused by whether or not identical molecules are involved, and it does not suggest that the physical properties of labeled and unlabeled molecules are different. To clearly demonstrate this point, let us analyze a dimerization reaction by a simple model.

Consider a dimerization reaction

$$\mathrm{A_2} \leftrightarrow \mathrm{A} + \mathrm{A} \qquad \text{with} \qquad K_{18} = \frac{k_{\mathrm{d},18}}{k_{\mathrm{a},18}} = \frac{[\mathrm{A}]^2}{[\mathrm{A_2}]} \tag{18}$$

and that when some A molecules are fluorescently labeled as in the manuscript

$$\mathrm{AA^*} \leftrightarrow \mathrm{A^*} + \mathrm{A} \qquad \text{with} \qquad K_{19} = \frac{k_{\mathrm{d},19}}{k_{\mathrm{a},19}} = \frac{[\mathrm{A}][\mathrm{A^*}]}{[\mathrm{AA^*}]} \tag{19}$$

We assert that the labeling does not alter any physical properties. So, the dissociation rate constants of reactions (18) and (19) are identical, $k_{\mathrm{d},18}$=$k_{\mathrm{d},19}$. We now apply the simple collision theory, a standard model in every physical chemistry textbook, to reveal the relationship of the association rate constants between the two reactions. The association rate constant of reaction (18) will be one half of that of reaction (19), $k_{\mathrm{a},18}$=$k_{\mathrm{a},19}$/2, because all the factors are the same except that the collision frequency between identical molecules (reaction (18)) is one half of that between different molecules (reaction (19)) under unit concentrations. Therefore, the equilibrium dissociation constant of reaction (18) (K_{18}) is twice that of reaction

(19) (K_{19}).

$$K_{18} = \frac{k_{\mathrm{d},18}}{k_{\mathrm{a},18}} = \frac{k_{\mathrm{d},19}}{k_{\mathrm{a},19}/2} = 2K_{19} \tag{20}$$

In the case of trimerization, it is hard to assess the relationship between Reactions (6) and (8) with a simple model, because the results will be dependent on specific reaction mechanisms. However, the same logic will lead to the same conclusions that K and K^* are different.

Supplementary note 3: Derivation of equilibrium constants of the formation of OmpC-Skp complexes

For the equilibrium

$$\mathrm{OmpC} \cdot \mathrm{Skp}_3 \overset{K_{\mathrm{D}}}{\leftrightarrow} \mathrm{OmpC} + 3\mathrm{Skp} \tag{21}$$

where the apparent dissociation constant K_{D} is the concentration of Skp at which half OmpC molecules are in the complex. The normalized event counts of the colocalized donor-acceptor pairs on the TIRF experiments (p) is fitted by

$$p = \frac{[\mathrm{Skp}]_0{}^{n_{\mathrm{Hill}}}}{K_{\mathrm{D}}^{n_{\mathrm{Hill}}} + [\mathrm{Skp}]_0{}^{n_{\mathrm{Hill}}}} \tag{22}$$

where $[\mathrm{Skp}]_0$ is the total concentration of the Skp in the solution and n_{Hill} is the Hill coefficient representing the cooperativity of the reaction. The normalized p against $[\mathrm{Skp}]_0$ was plotted and fitted to obtain K_{D} and n_{Hill} (Fig. 3a in the main text).

For the equilibrium

$$\mathrm{OmpC}\cdot(\mathrm{Skp_3})_2 \overset{K_\mathrm{D}'}{\leftrightarrow} \mathrm{OmpC}\cdot\mathrm{Skp_3}+\mathrm{Skp_3} \tag{23}$$

where $K_\mathrm{D}' = \frac{[\mathrm{OmpC\cdot Skp_3}][\mathrm{Skp_3}]}{[\mathrm{OmpC\cdot(Skp_3)_2}]}$ is the respective dissociation constant. The fraction of $[\mathrm{OmpC\cdot(Skp_3)_2}]$ over $[\mathrm{OmpC\cdot Skp_3}]+[\mathrm{OmpC\cdot(Skp_3)_2}]$ (f) represented by the respective smFRET peak areas is derived to be

$$f = \frac{[\mathrm{Skp}]_0}{3K_\mathrm{D}'+[\mathrm{Skp}]_0} \tag{24}$$

The fitting of the smFRET peak areas positioned at 0.3 in Supplementary Fig. S13 was carried out to obtain f. Then, the normalized f as a function of $[\mathrm{Skp}]_0$ was plotted and fitted to obtain K_D' (Fig. 4d in the main text).

Supplementary Figures

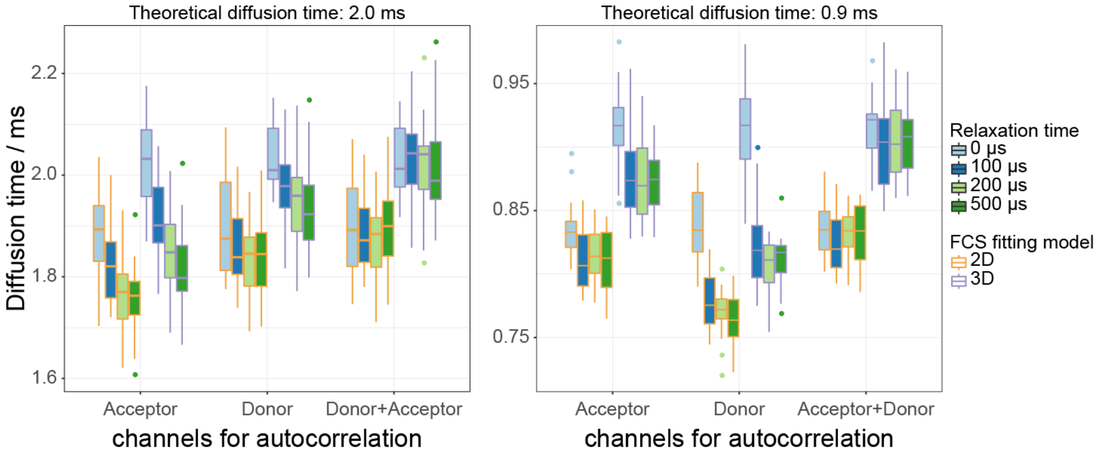

Supplementary Figure S1 The influence of the relaxation time and the fashion of donor/acceptor correlations on the extracted diffusion time in the simulated pscFCS curves.

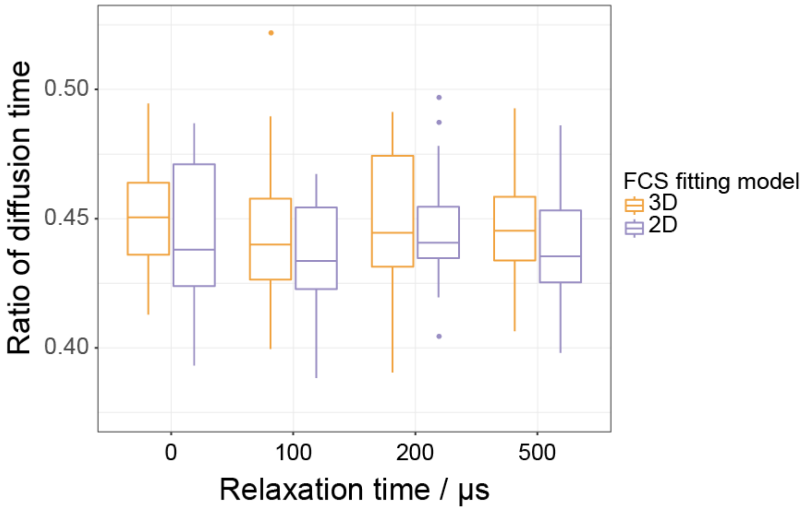

Supplementary Figure S2 The comparison of the 2D1R model to the 3D1R model on the ratio of extracted diffusion times of two species with the theoretical diffusion time of 0.9 ms and 2.0 ms respectively. The pscFCS curves were generated by adding the donor and acceptor traces together.

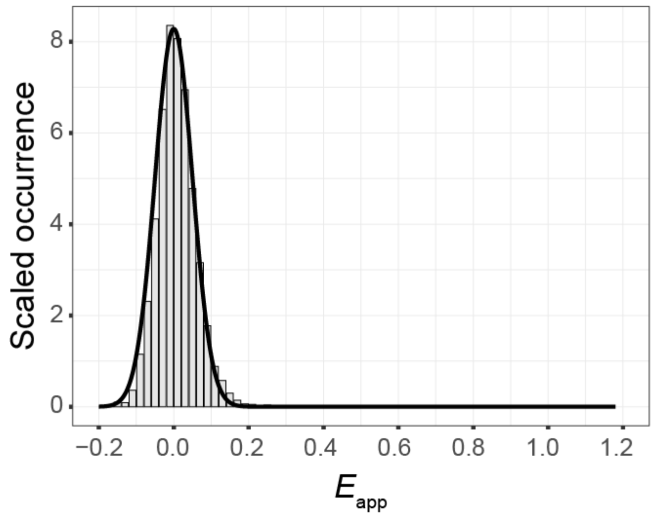

Supplementary Figure S3 The smFRET histogram of Cy3B. The histogram only has a zero-efficiency peak due to the absence of the acceptor dyes. The smFRET efficiencies ranging from -0.1 to 0.1 were included to calculate the pscFCS curves.

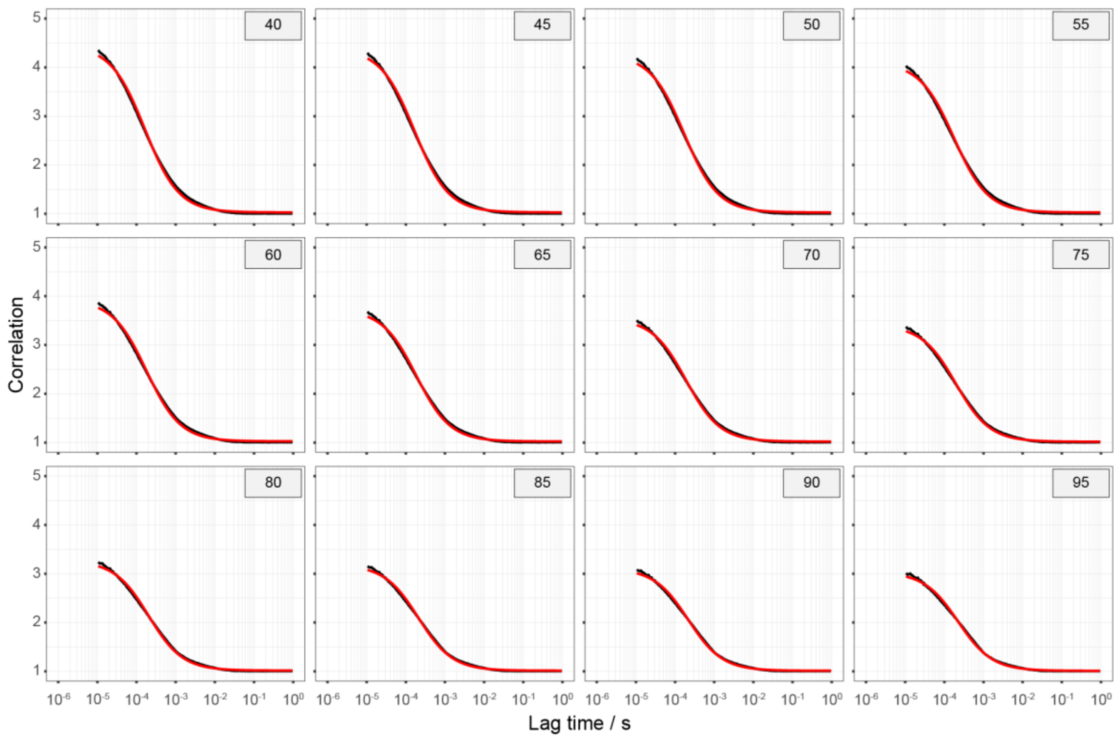

Supplementary Figure S4 The pscFCS curves of Cy3B starting from 10 μs and the respective 2D fit (with the smFRET efficiencies ranging from -0.1 to 0.1) at different peak

thresholds. The black lines and red lines represent the experimental data and fitted curves, respectively. The extracted apparent diffusion times were plotted and fitted in Fig. 1d in the main text.

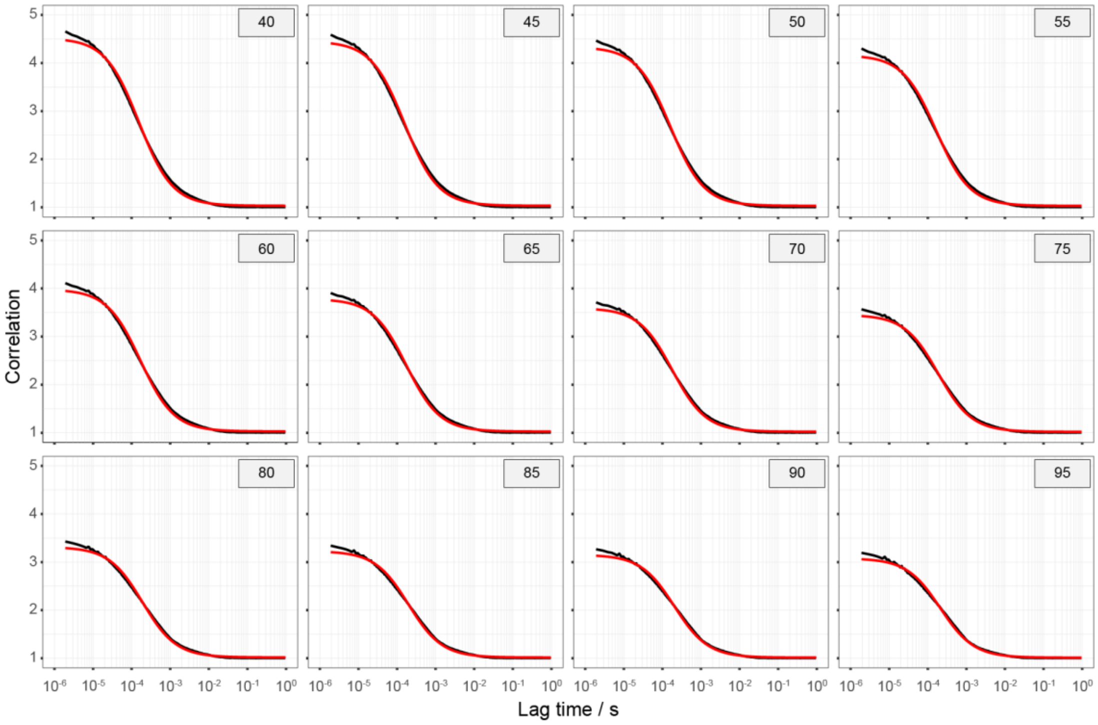

Supplementary Figure S5 The pscFCS curves of Cy3B starting from 1 μs and the respective 2D fit (with the smFRET efficiencies ranging from -0.1 to 0.1) at different peak thresholds. The black lines and red lines represent the experimental data and fitted curves, respectively.

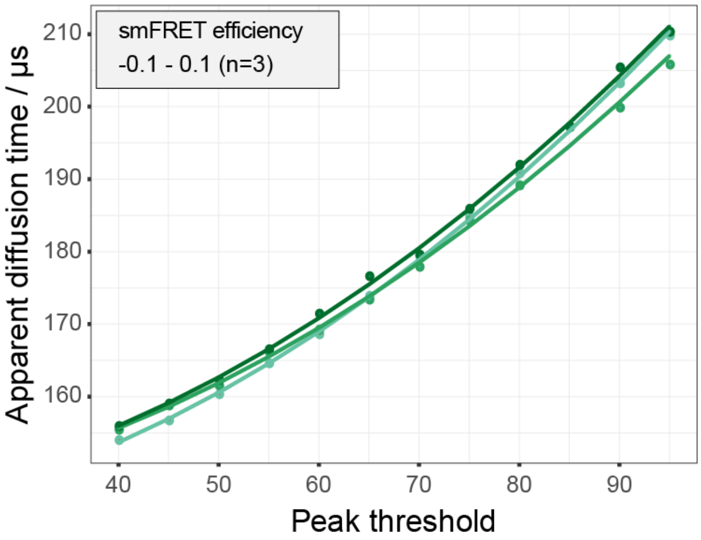

Supplementary Figure S6 The plot of the apparent diffusion time extracted from the 2D fit of the 1 µs-started FCS curves against the peak threshold for Cy3B. The unbiased diffusion time was 143±2 µs. The dots and lines represent the experimental data and fitted curves, respectively. Data are shown of three independent experiments.

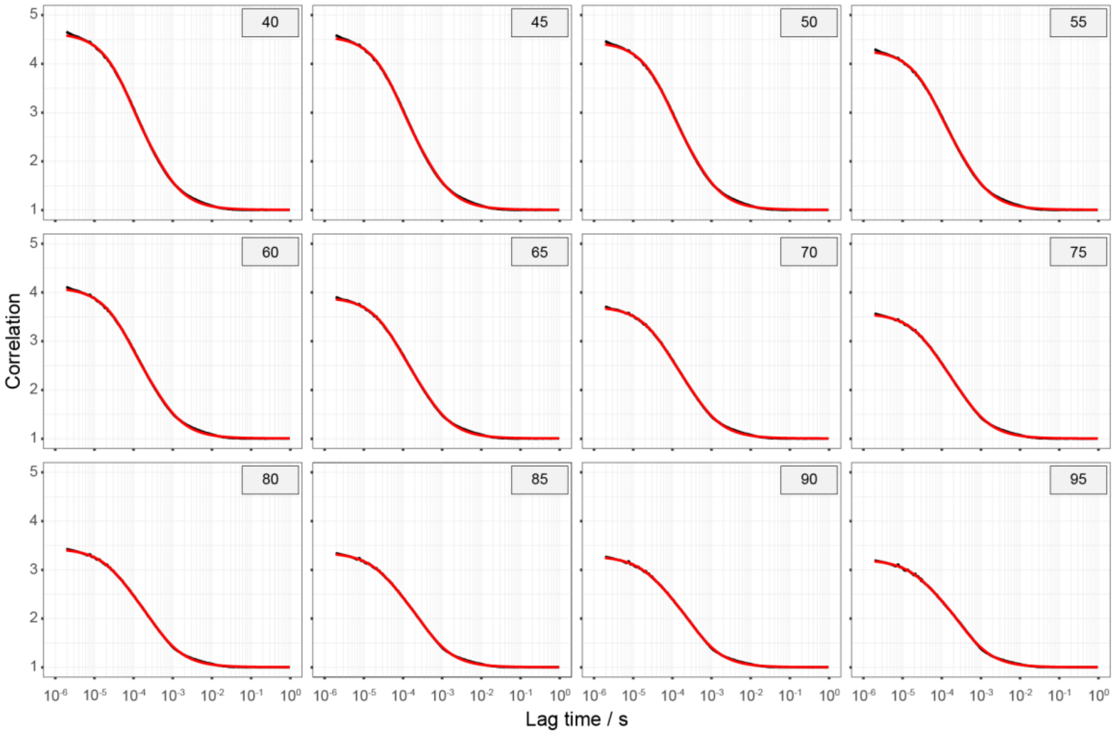

Supplementary Figure S7 The pscFCS curves of Cy3B starting from 1 µs and the respective

2D1R fit (with the smFRET efficiencies ranging from -0.1 to 0.1) at different peak thresholds. The black lines and red lines represent the experimental data and fitted curves, respectively.

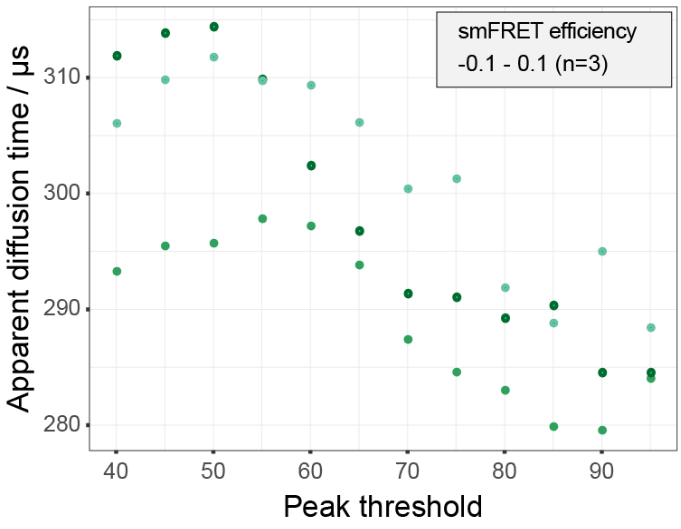

Supplementary Figure S8 The plot of the apparent diffusion time extracted from the 2D1R fit of the 1 µs-started FCS curves of Cy3B against the peak threshold. The data were irregular and could not be described by equation (4). The dots represent the experimental data. Data are shown of three independent experiments.

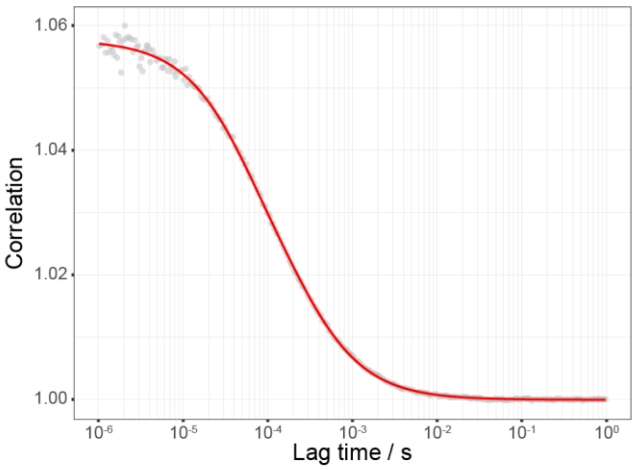

Supplementary Figure S9 An example of the conventional FCS data and the respective fit.

The sample contained 5 nM Cy3B. The grey dots and the red line represent the experimental data and the fitted curve, respectively.

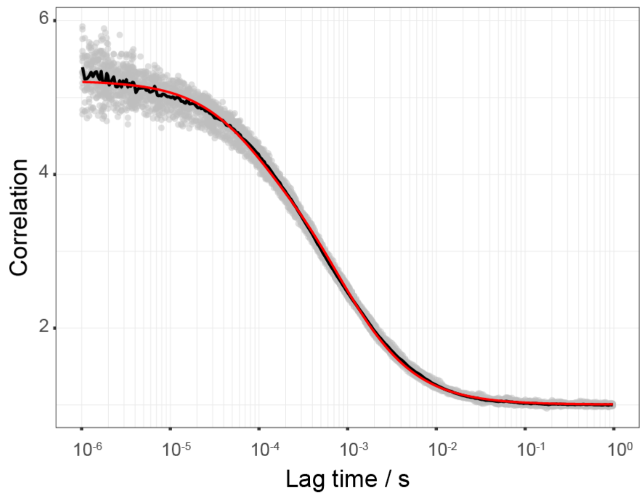

Supplementary Figure S10 An example of the FCS data and respective fit for the Skp self-trimerization. The sample contained 0.4 nM Skp D128C-Cy3B and 140 nM Skp. The grey dots, the black line and the red line represent the experimental data, the data after averaging and the fitted curve, respectively.

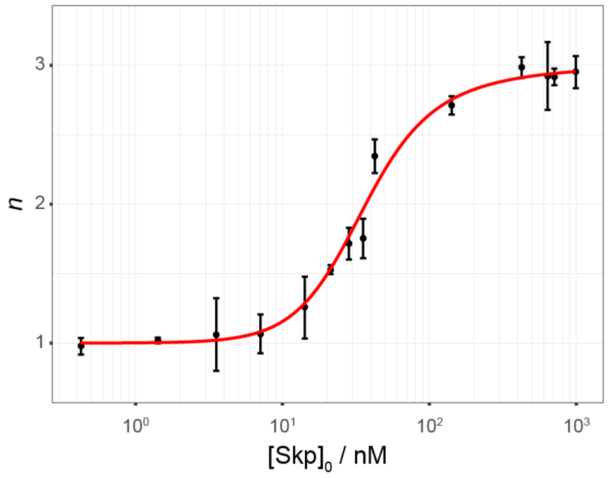

Supplementary Figure S11 The titration curve of n as a function of $[Skp]_0$. The black dots and the red line represent the experimental data and the fitted curve, respectively. Data are

shown as mean±s.d. of three independent experiments. The K and the K^* were obtained simultaneously by fitting equation (11), (14) and (15).

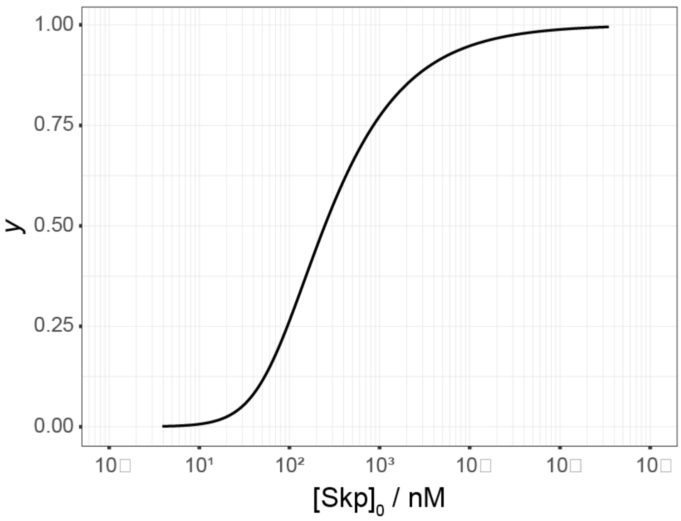

Supplementary Figure S12 The deduced titration curve of the population of the wild-type Skp molecules in the trimer form (y) as a function of $[Skp]_0$. The curve is drawn according to equation (17) and the fitted K.

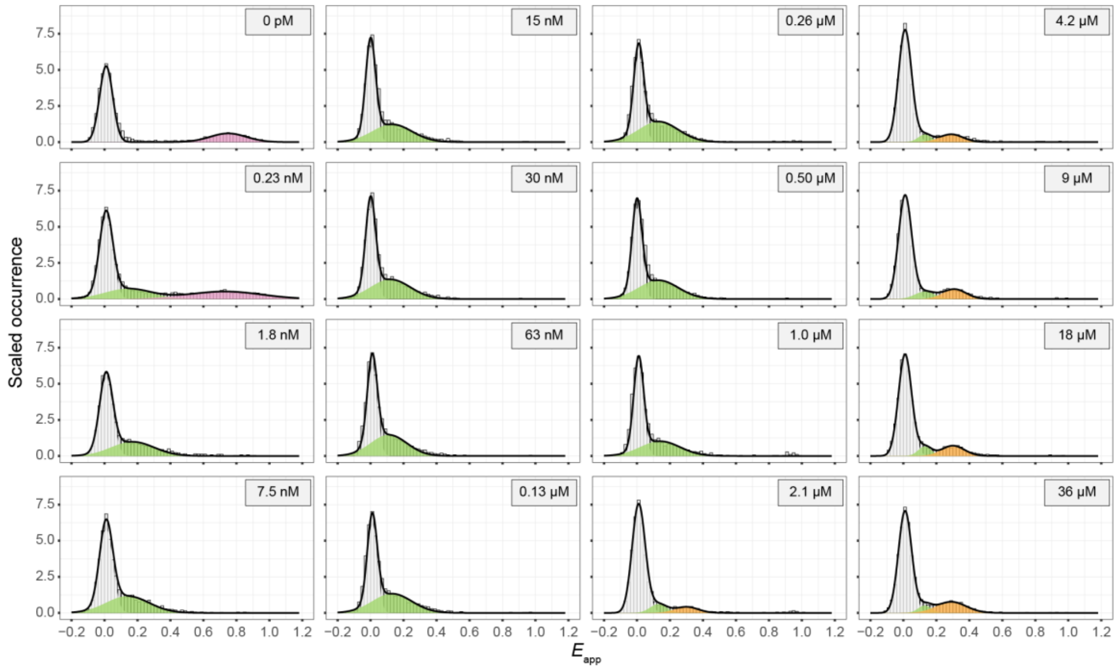

Supplementary Figure S13 The intramolecular smFRET histograms of 50 pM OmpC G8C-D335C in Skp of different concentrations. The unbound-OmpC, OmpC·Skp_3 and OmpC·$(Skp_3)_2$ are colored in pink, green and amber, respectively. The zero-efficiency peak resulted from missing or inactivated acceptors is colored in gray. All histograms were normalized and fitted by the Gaussian distributions.

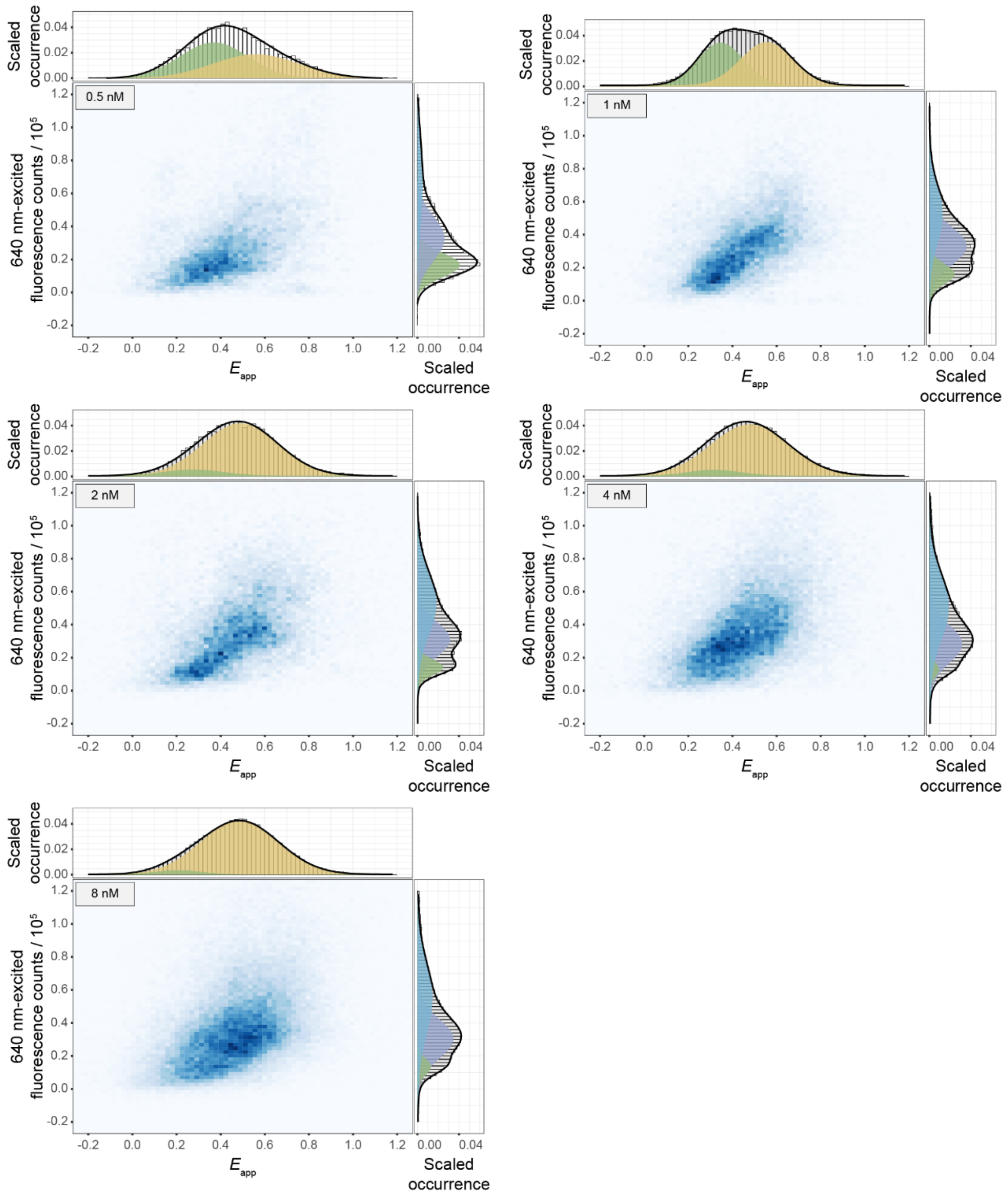

Supplementary Figure S14 The 2D histograms of the immobilized OmpC G8C-AF555 in the Skp D128C-AF647 solution of different concentrations. The histograms on the fluorescence counts dimension exhibited three peaks respectively positioned at 0.15×10^5 (green), 0.31×10^5 (indigo) and 0.56×10^5 (azure), demonstrating that OmpC bound more than one Skp monomers. The histograms on the smFRET efficiency dimension could be fitted by two peaks respectively positioned at 0.30 (green) and 0.51 (amber), suggesting that the

intermolecular E_{app} was close when OmpC bound different numbers of Skp monomers. All histograms were normalized and fitted by the Gaussian distributions.

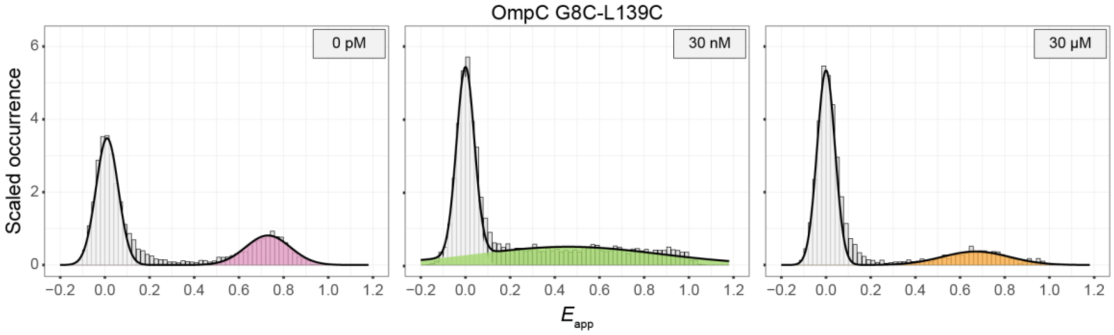

Supplementary Figure S15 The smFRET histograms of 50 pM OmpC G8C-L139C in Skp of 0 pM, 30 nM and 30 μM. The unbound-OmpC, OmpC·Skp_3 and OmpC·$(Skp_3)_2$ are colored in pink, green and amber, respectively. The zero-efficiency peak resulted from missing or inactivated acceptors is colored in gray. All histograms were normalized and fitted by the Gaussian distributions.

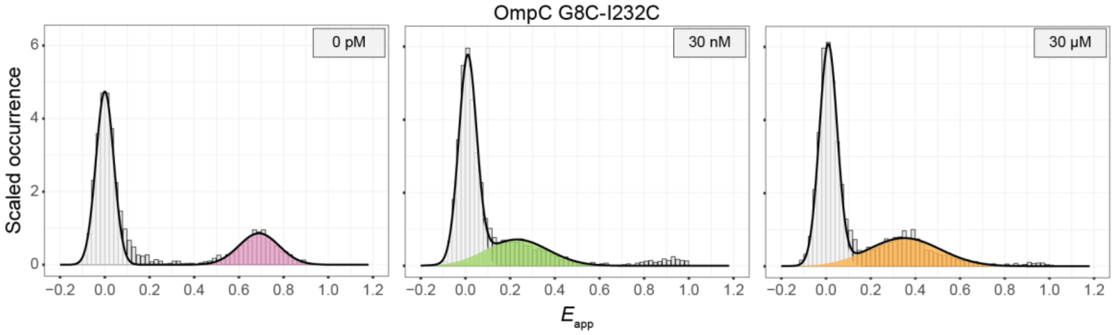

Supplementary Figure S16 The smFRET histograms of 50pM OmpC G8C-I232C in Skp of 0 pM, 30 nM and 30 μM. The unbound-OmpC, OmpC·Skp_3 and OmpC·$(Skp_3)_2$ are colored in pink, green and amber, respectively. The zero-efficiency peak resulted from missing or inactivated acceptors is colored in gray. All histograms were normalized and fitted by the

Gaussian distributions.

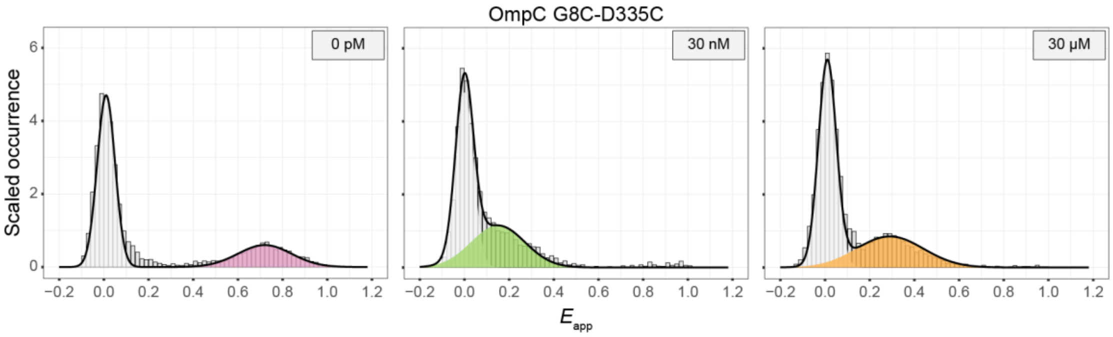

Supplementary Figure S17 The smFRET histogram of 50 pM OmpC G8C-D335C in Skp of 0 pM, 30 nM and 30 μM. The unbound-OmpC, OmpC·Skp_3 and OmpC·$(Skp_3)_2$ are colored in pink, green and amber, respectively. The zero-efficiency peak resulted from missing or inactivated acceptors is colored in gray. All histograms were normalized and fitted by the Gaussian distributions.

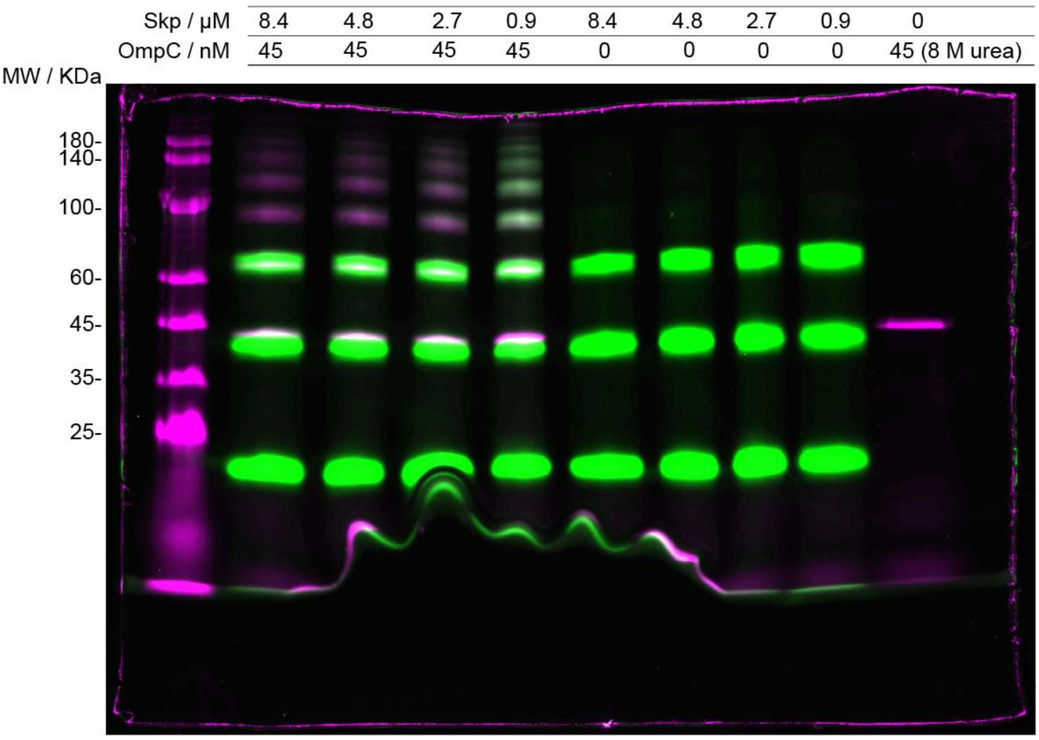

Supplementary Figure S18 The amine-crosslinked SDS-PAGE of Skp D128C-Cy3B and OmpC G8C-AF647. The crosslinking efficiency decreased with the increase of the Skp concentration because the excessive Skp consumed the crosslinking reagent.

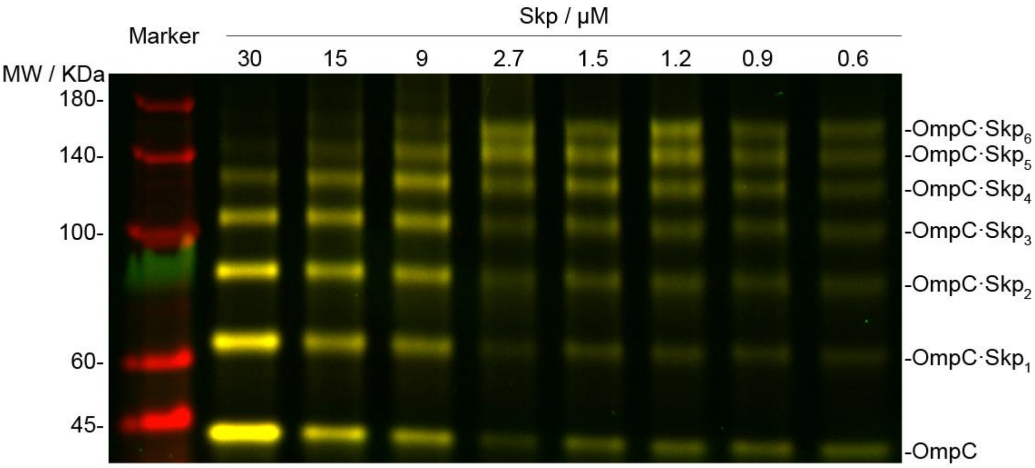

Supplementary Figure S19 The amine-crosslinked SDS-PAGE of Skp and intramolecular labelled OmpC G8C-D335C. The gel bands were assigned to the crosslinked OmpC·Skp$_n$

complexes according to their molecular weight. The crosslinking efficiency decreased with the increase of the Skp concentration because the excessive Skp consumed the crosslinking reagent.

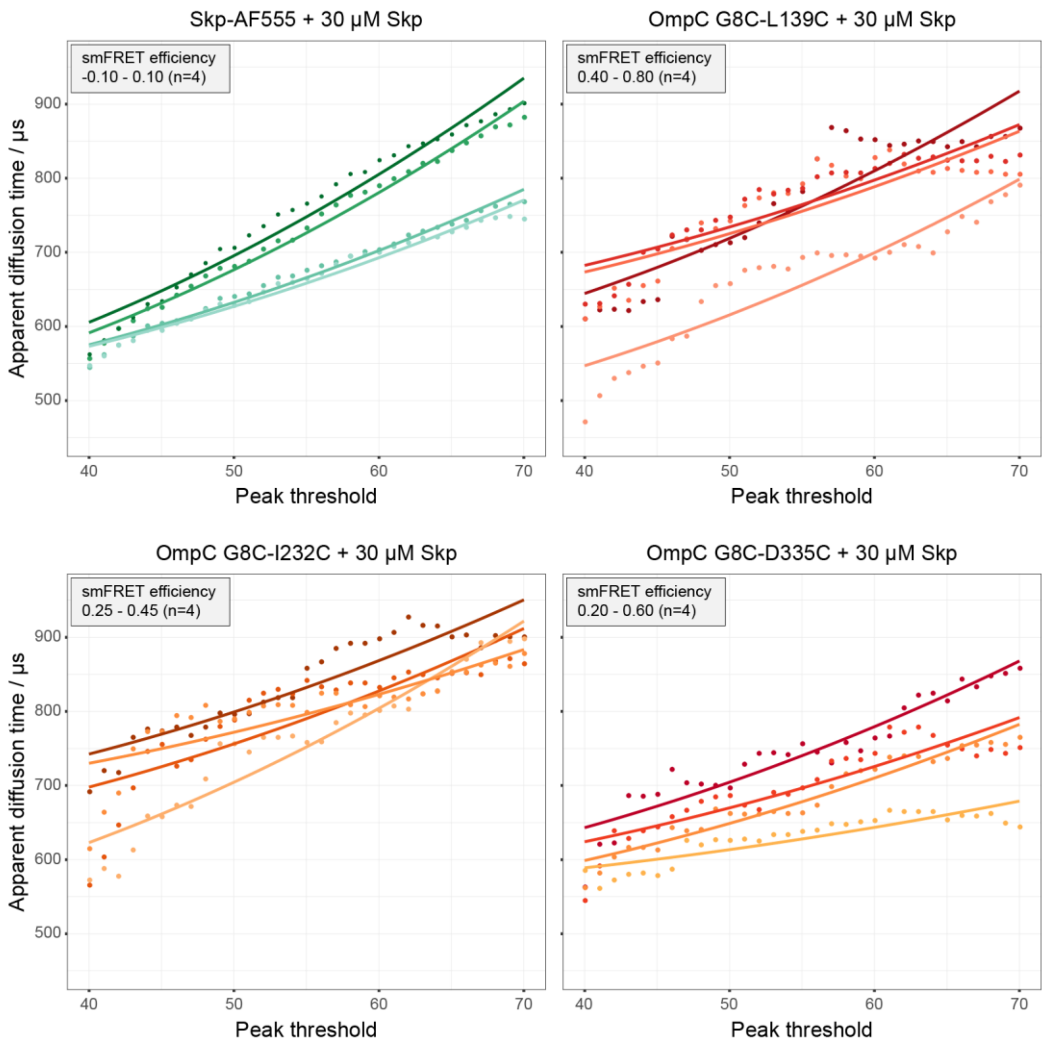

Supplementary Figure S20 The pscFCS treatment of different dual-labelled OmpC mutants in Skp of 30 μM. Skp D128C-AF555 in Skp of 30 μM was used as a reference. The calculated results are listed in Supplementary Table S2.

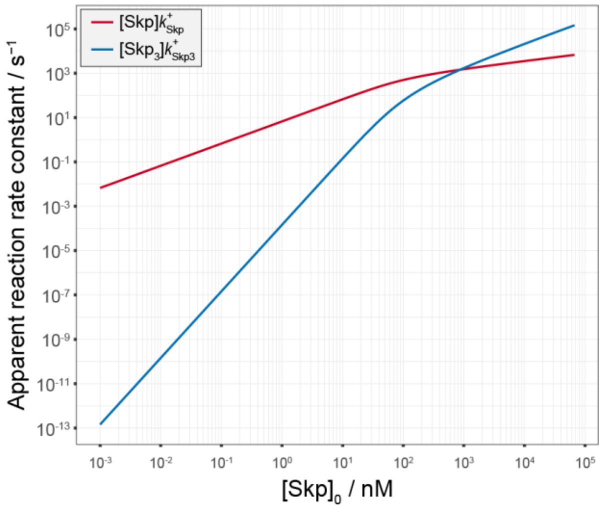

Supplementary Figure S21 The deduction of apparent reaction rates of Skp and Skp$_3$ to OmpC. The apparent reaction rate constant is simulated according to the estimated association rate constants from the molecule radius and the fitted dissociation constants K of the self-trimerization between Skp and Skp$_3$.

Supplementary Tables

Supplementary Table S1 Estimation of molecular weight and number of linked DSS molecules in intermolecular labelled OmpC and Skp in fluorescent SDS-PAGE

**Molecules**	**MW range (kD)**	**Theoretical MW (kD)**	**ΔMW (kD)**	**#DSS (Total)**	**#DSS (OmpC+Skp×n)[a]**
OmpC·Skp_6	189 - 198	154	40	108	10+16×6
OmpC·Skp_5	161 - 176	135	33	90	10+16×5
OmpC·Skp_4	134 - 141	116	22	59	10+12×4
OmpC·Skp_3	112 - 121	97	19	53	10+14×3
OmpC·Skp_2	90 - 93	78	14	37	10+13×2
OmpC·Skp_1	65 - 71	59	10	27	10+17×1
Skp_3	65 - 71	57	10	27	0+9×3
OmpC	43 - 44	40	3.5	10	10+0
Skp_2	41 - 42	38	3.6	10	0+5×2
Skp	16 - 20	19	-0.9	-3	0+0

a Proposed possible numbers of DSS molecules among protein molecules

Supplementary Table S2 Hydrodynamic radius of different molecules derived from the pscFCS treatment

**Subpopulation**	**Labelling sites**	**Diffusion time / µs**	**Radius / nm**
Skp_3	Skp D128C	459±19	3.3±0.1[a]
OmpC·Skp_3	OmpC G8C-D335C	549±27	3.9±0.2
OmpC·$(Skp_3)_2$	OmpC G8C-L139C	527±77	3.8±0.6
OmpC·$(Skp_3)_2$	OmpC G8C-I232C	592±81	4.3±0.6
OmpC·$(Skp_3)_2$	OmpC G8C-D335C	533±16	3.8±0.1

a Taken from reference 25 in the main text.
